# Unavoidable future increase in West Antarctic ice-shelf melting over the twenty-first century

**DOI:** 10.1038/s41558-023-01818-x

**Published:** 2023-10-23

**Authors:** Kaitlin A. Naughten, Paul R. Holland, Jan De Rydt

**Affiliations:** 1 British Antarctic Survey, Cambridge, UK; 2 Northumbria University, Newcastle upon Tyne, UK

**Keywords:** Climate and Earth system modelling, Projection and prediction, Cryospheric science, Physical oceanography, Physical oceanography

## Abstract

Ocean-driven melting of floating ice-shelves in the Amundsen Sea is currently the main process controlling Antarctica’s contribution to sea-level rise. Using a regional ocean model, we present a comprehensive suite of future projections of ice-shelf melting in the Amundsen Sea. We find that rapid ocean warming, at approximately triple the historical rate, is likely committed over the twenty-first century, with widespread increases in ice-shelf melting, including in regions crucial for ice-sheet stability. When internal climate variability is considered, there is no significant difference between mid-range emissions scenarios and the most ambitious targets of the Paris Agreement. These results suggest that mitigation of greenhouse gases now has limited power to prevent ocean warming that could lead to the collapse of the West Antarctic Ice Sheet.

## Abstract (web summary)

The authors use a regional ocean model to project ocean-driven ice-shelf melt in the Amundsen Sea. Already committed rapid ocean warming drives increased melt, regardless of emission scenario, suggesting extensive ice loss from West Antarctica.

## Main

**Fig. 1:**
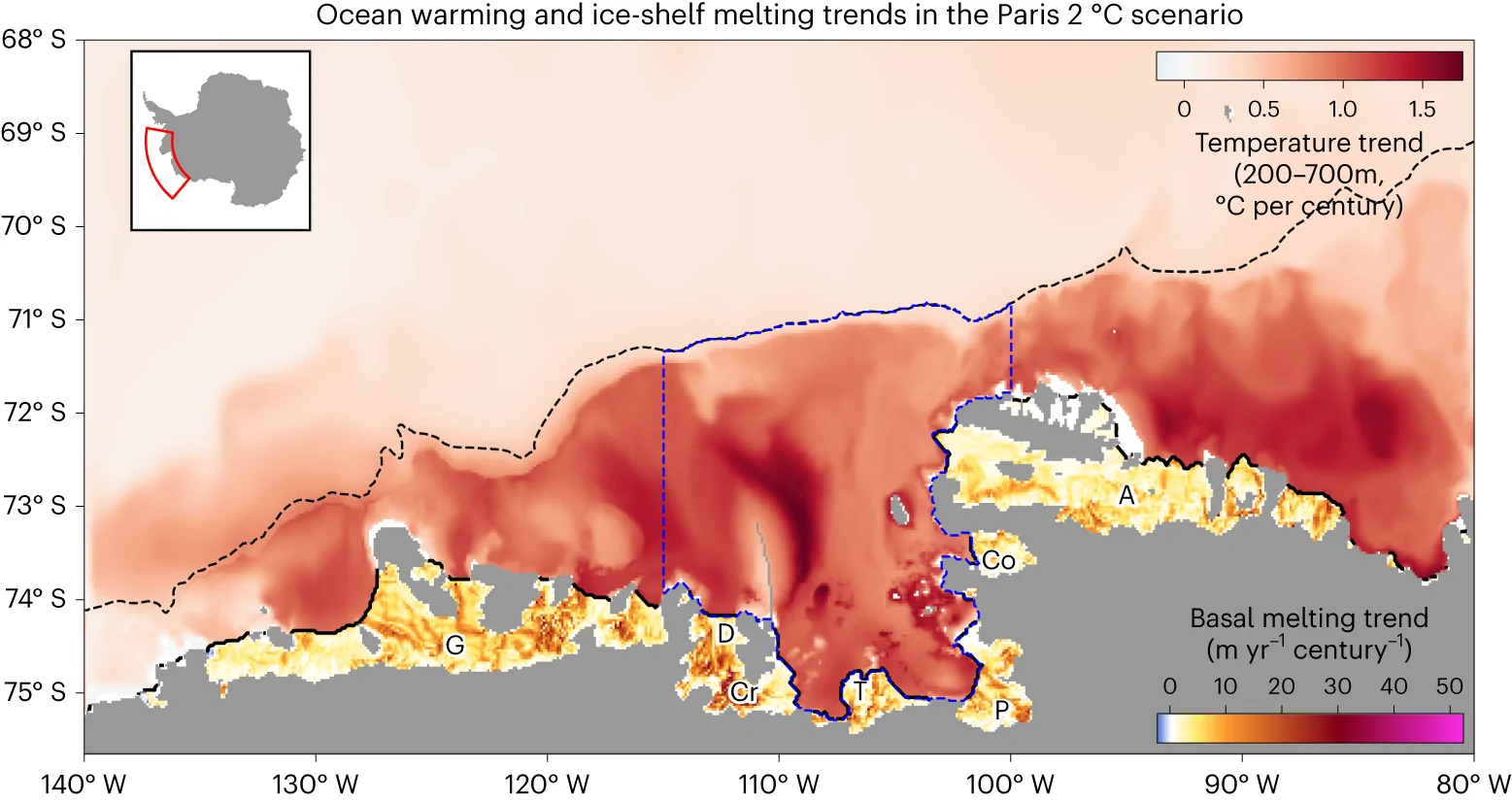
Map of ensemble mean trends in ocean temperature and ice-shelf basal melting in the Paris 2 °C scenario. Temperature is averaged over the depth range 200–700 m. Trends are calculated at each point using annually averaged fields from 2006–2100. White regions indicate no significant trend. The Amundsen Sea region visualized here (latitude–longitude projection) is outlined in red in the inset map of Antarctica (polar stereographic projection). The black dashed line shows the 1,750 m depth contour of the continental shelf break and the blue dashed line outlines the continental shelf region used for analysis. Labels denote ice shelves (G, Getz; D, Dotson; Cr, Crosson; T, Thwaites; P, Pine Island; Co, Cosgrove; A, Abbot).

The West Antarctic Ice Sheet (WAIS) is losing mass and is Antarctica’s largest contributor to sea-level rise^1^. This ice loss is driven by interactions with the Southern Ocean^2^, particularly the Amundsen Sea region of the continental shelf seas (Fig. 1). Enhanced basal melting of ice shelves, the floating extensions of the ice sheet, has reduced their buttressing and caused upstream glaciers to accelerate their flow towards the ocean^3^. Continued trends in ice-shelf melting have the potential to cause irreversible retreat of the WAIS glaciers^4^, which together contain enough ice to raise global mean sea-level by 5.3 m (ref. ^5^).

Previous modelling^6^ found that the Amundsen Sea probably warmed in response to atmospheric changes over the twentieth century, providing a viable explanation for WAIS mass loss. Observations cannot be used to detect such a long-term ocean warming trend in this region, as data collection only began in 1994^7^, and the warming trend exhibits strong decadal variability^8^. Regardless of the existence or magnitude of historical trends, if the Amundsen Sea experiences further warming over the twenty-first century, the outlook for the WAIS will only become more grave.

It has been hypothesized that Amundsen Sea warming will respond to future climate change and may be amenable to mitigation by reducing greenhouse gas emissions^6,9,10^. However, this hypothesis has not been adequately tested. Existing future projections of ice-shelf basal melting are generally not reliable in the Amundsen Sea, a region which is frequently biased cold or poorly resolved in the underlying ocean models^11,12^. Reference ^13^ produced the first future projections using a regional model of the Amundsen Sea, which simulated an increase in basal melting. However, this study only considered a single forcing scenario, the worst-case scenario of extreme fossil fuel use, and did not account for internal climate variability.

To enable policymakers and global communities to appropriately respond to sea-level rise, it is necessary to analyse not just the worst-case scenario for fossil fuel use, but also the best-case scenario, as well as scenarios in between. A thorough investigation of scenario dependence will begin to address highly policy-relevant questions: How much sea-level rise from the WAIS is now unavoidable and must be adapted to? How much ice loss does the international community still have control over, by reducing greenhouse gas emissions? How long does it take before the different scenarios diverge and the results of chosen climate policies become clear? Finally, to what degree will future sea-level rise be determined by the pathway of internal climate variability versus anthropogenic forcing?

## Approach

**Extended Data Fig. 1:**
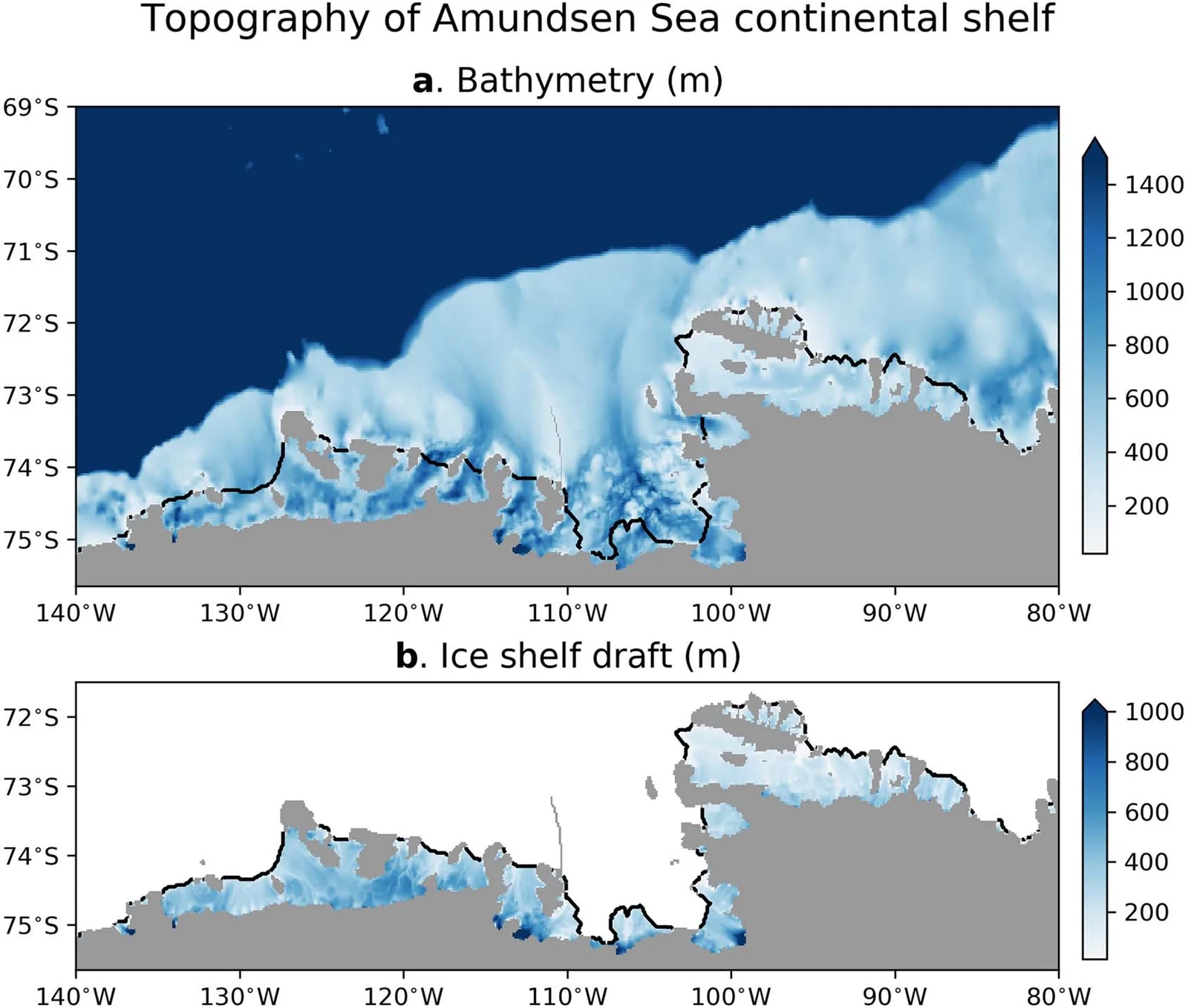
Topography of the continental shelf in the model. The full model domain extends north to 62°S. **(a)** Bathymetry, **(b)** ice-shelf draft, both in metres where positive indicates downward (note different colour scales).

**Extended Data Fig. 2:**
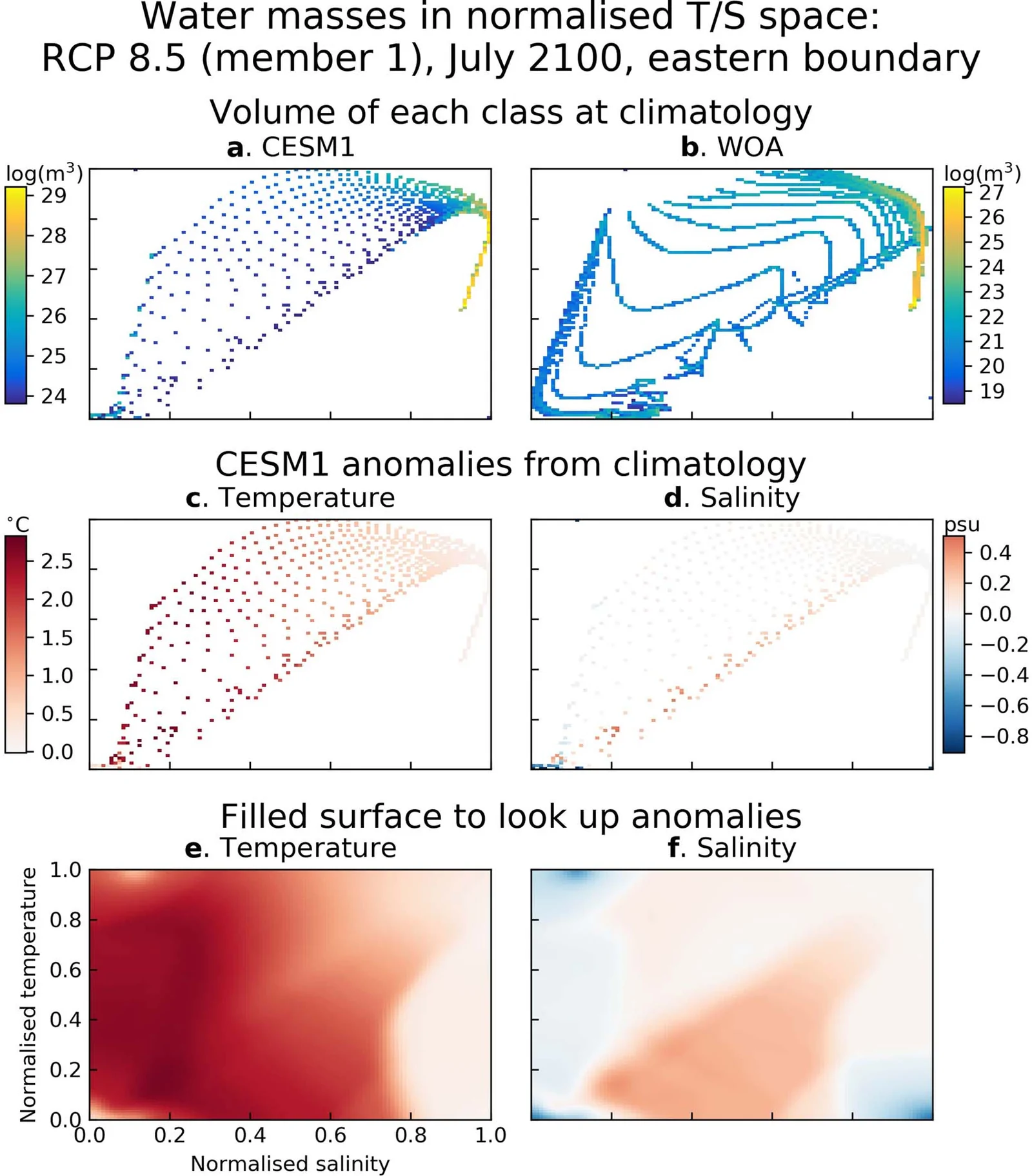
Intermediate steps in bias correction of temperature and salinity. This example shows the eastern model boundary (80°W) for July 2100 in RCP 8.5 (first ensemble member). Data is expressed in normalised T/S space, where each axis ranges from 0 (coldest or freshest water on boundary) to 1 (warmest or saltiest) and is divided into 100 bins. (**a**,**b**) Volume of water in each bin for the July CESM1 climatology (1998-2017, mean of 40-member LENS ensemble^14^) and World Ocean Atlas 2018 climatology^34^ respectively. Bins with zero volume are masked in white. The 20-year period 1998-2017 was chosen for CESM1 to approximately align with the observations informing WOA. The two plots have different colour scales because the different horizontal resolutions of the two products affect the grid cell volumes; however, the WOA volume is not used in the bias correction and is only shown for illustrative purposes. (**c**, **d**) CESM1 temperature and salinity anomalies for the specific month (July 2100, RCP 8.5, member 1) with respect to the July climatology in **(a)**. The volume-weighted mean anomaly is shown for each bin. (**e**,**f**) As in (**c**,**d**) but with missing values filled in, creating a complete surface from which to look up anomalies for each WOA bin in **(b)**.

**Extended Data Fig. 3:**
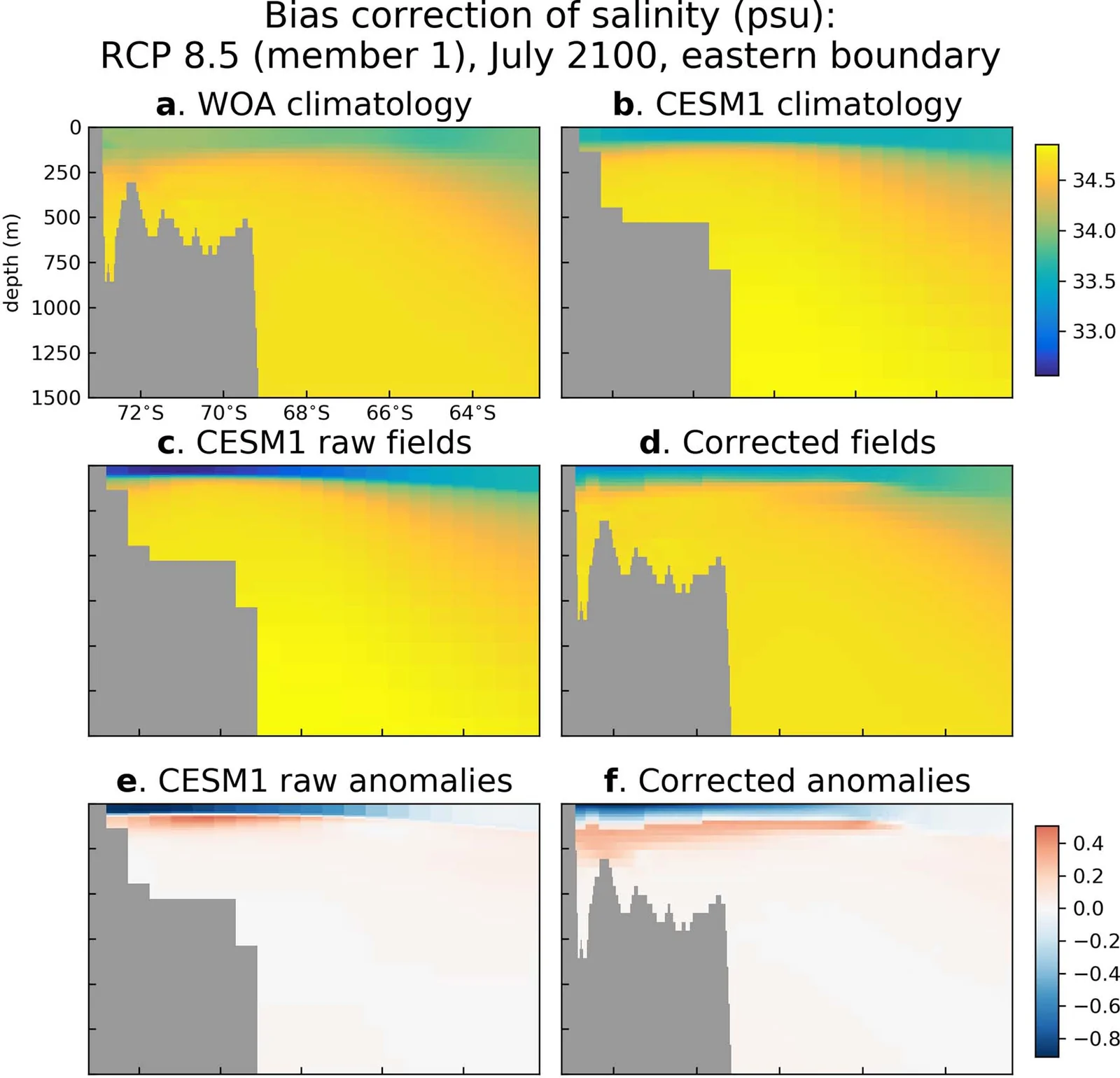
Bias correction of salinity in physical space (depth vs latitude). The same example month as in Extended Data Fig. 2 is shown, for the eastern boundary zoomed into the depth range above 1500 m. **(a)** WOA climatology for July, interpolated to MITgcm grid; **(b)** CESM1 present-day climatology for July, on original CESM1 grid; **(c)** raw CESM1 output for July 2100; **(d)** final corrected salinity field; **(e)** raw CESM1 anomalies (c minus b); **(f)** corrected CESM anomalies (d minus a).

Here we present a suite of future projections simulated by a regional ocean model of the Amundsen Sea, including sea ice and ice-shelf cavities (Methods and Extended Data Fig. 1). This configuration of the Massachusetts Institute of Technology general circulation model (MITgcm) ice–ocean model, forced by atmospheric output from the Community Earth System Model (CESM1) climate model, has been previously published and validated for present-day and twentieth-century simulations^6^. It is not coupled to an ice-sheet model, meaning the ice-shelf geometry does not change over time, although the model does simulate ice-shelf basal melting and the associated heat and freshwater fluxes. The atmospheric forcing is bias corrected following ref. ^6^. New to this study is a bias-correction method for CESM1 ocean fields on the open lateral boundaries of MITgcm (Methods, and Extended Data Figs. 2 and 3). This approach allows the ocean boundary conditions to evolve over time, considering the effects of remote changes in water masses in a manner consistent with the atmospheric forcing.

We simulate five core scenarios, one historical and four future, following CESM1 climate change experiments^14–16^. The historical scenario (1920–2005) follows observed external forcing, both anthropogenic and natural. The Paris 1.5 °C and Paris 2 °C scenarios (2006–2100) stabilize global mean temperature change at the given thresholds relative to pre-industrial conditions, following the goals of the Paris Agreement. Finally, the RCP 4.5 (2006–2080) and RCP 8.5 (2006–2100) scenarios follow Representative Concentration Pathways for future anthropogenic forcing, assuming medium and high fossil fuel use, respectively. It should be noted that limiting the global temperature rise to 1.5 °C is now considered unlikely given current levels of warming^17^; conversely, the RCP 8.5 scenario is considered unrealistically extreme given available fossil fuel reserves^18^. This suite of four scenarios therefore robustly bounds, on both ends, all likely future pathways of climate mitigation.

Two additional scenarios with climatological ocean boundary conditions (Historical Fixed BCs, RCP 8.5 Fixed BCs) allow us to quantify the impact of remote water mass changes. Ensembles of 5–10 members, each with a different realization of internal climate variability in CESM1, are run for each scenario (Extended Data Table 1). The use of ensembles is particularly important for the Amundsen Sea, where Pacific modes of variability have a strong influence and have probably contributed to historical trends^9,10^.

## Scenario dependence of warming

All scenarios exhibit significant and widespread future warming of the Amundsen Sea and increased melting of its ice shelves. The spatial distribution of trends is shown in Fig. 1 for the Paris 2 °C scenario. Here we analyse mid-depth temperature (200–700 m mean), the water which directly affects the ice-shelf cavities (Extended Data Fig. 1). Indeed, trends in mid-depth temperature significantly correlate with trends in ice-shelf basal mass loss (Extended Data Table 2). In reality, basal mass loss will also depend on other factors we cannot account for in our simulations, such as changes in ice-shelf geometry.

**Fig. 2:**
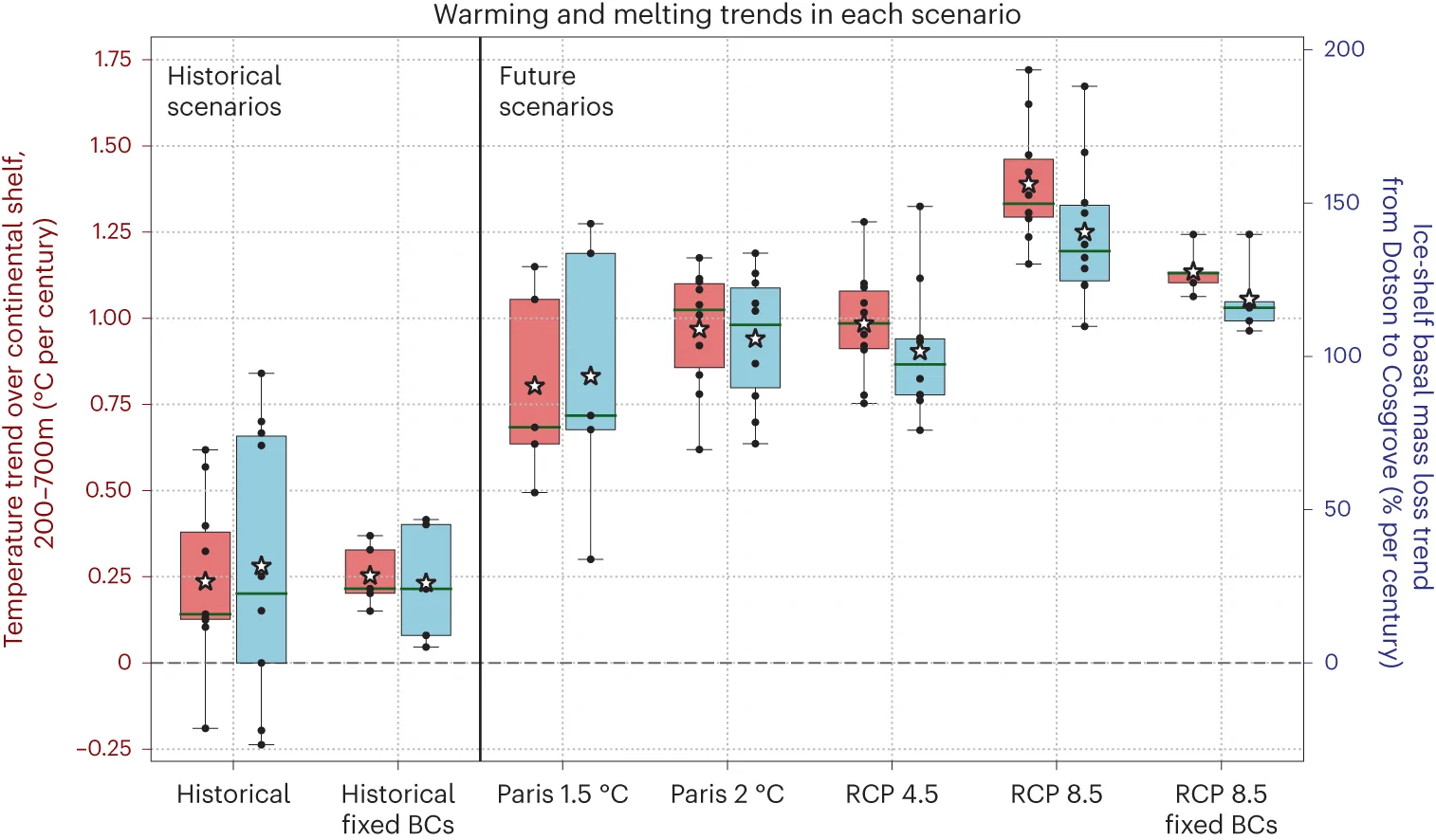
Boxplot of trends in ocean temperature and ice-shelf basal mass loss for each scenario. Ocean temperature trends are plotted in red (left axis); ice-shelf basal mass loss trends in blue (right axis). The scenarios are described in Extended Data Table 1; note different time spans and ensemble sizes (*n* = 5 for Paris 1.5 °C and the Fixed BCs scenarios, and *n* = 10 for all others). Temperature is averaged over the continental shelf and the depth range 200–700 m. Basal mass loss is integrated over the ice shelves between Dotson and Cosgrove inclusive and expressed as a percentage of the 1920–1949 historical ensemble mean. Both variables are smoothed with a 2-yr running mean before computing trends. Each scenario shows the ensemble mean (white stars), median (green lines), 25–75% range (boxes), full ensemble range (whiskers) and individual trends (black dots).

**Extended Data Fig. 4:**
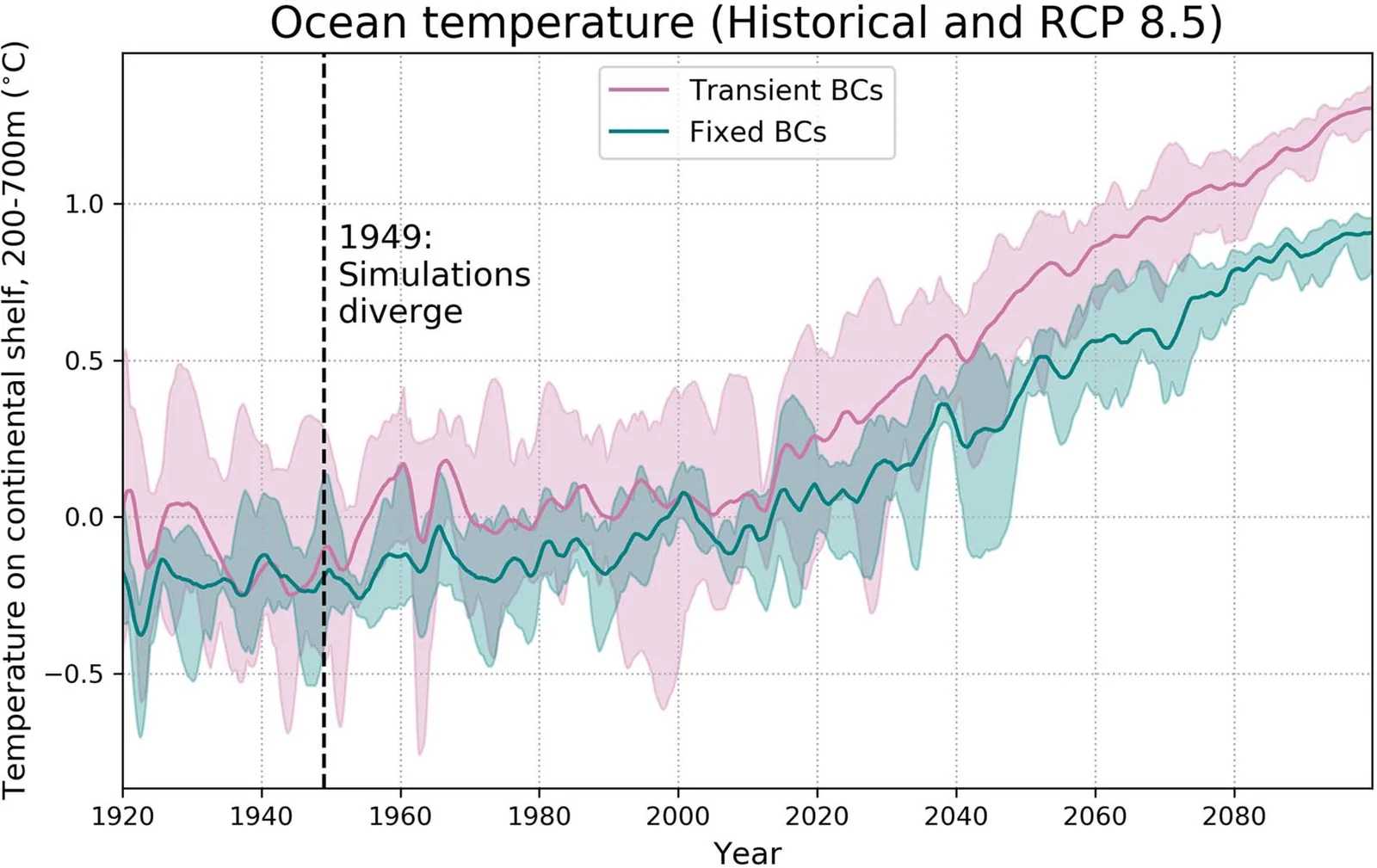
Timeseries of ocean temperature, comparing transient boundary conditions with fixed boundary conditions. All conventions are as in Fig. 3, for the Historical scenario moving into RCP 8.5.The dashed black line shows 1949, the year at which Historical diverges from Historical Fixed BCs (see Methods). For each scenario, the solid line shows the ensemble mean, and the shaded region the full ensemble range.

The trends in each scenario are compared using a boxplot in Fig. 2. Future warming and melting are markedly stronger than historical trends, with ensemble mean future warming trends ranging from 0.8 to 1.4 °C per century (Extended Data Table 1) compared with the historical mean of 0.25 °C per century. Even under the most ambitious mitigation scenario, Paris 1.5 °C, the Amundsen Sea warms three times faster than in the twentieth century. Comparison with the Fixed BCs simulations shows that local atmospheric changes are the main driver of Amundsen Sea warming, with remote ocean forcing playing a non-negligible secondary role (Supplementary Discussion 1 and Extended Data Fig. 4).

We also find a relatively large ensemble spread: future warming trends can vary by a factor of two depending on the phasing of internal climate variability. Nonetheless, the individual warming trends are significant for every ensemble member of every future scenario. By comparing the distributions of trends between two ensembles, we can determine whether different scenarios are distinct in terms of Amundsen Sea warming (Methods). The Paris 1.5 °C, Paris 2 °C and RCP 4.5 trends are all statistically indistinguishable, assessed in any combination, for both warming and melting. Only RCP 8.5, the most extreme scenario, is distinct from the others. This result suggests that climate mitigation has limited power to prevent ocean warming which controls sea-level rise from the WAIS and that internal climate variability presents a larger source of uncertainty than future greenhouse gas emissions.

**Fig. 3:**
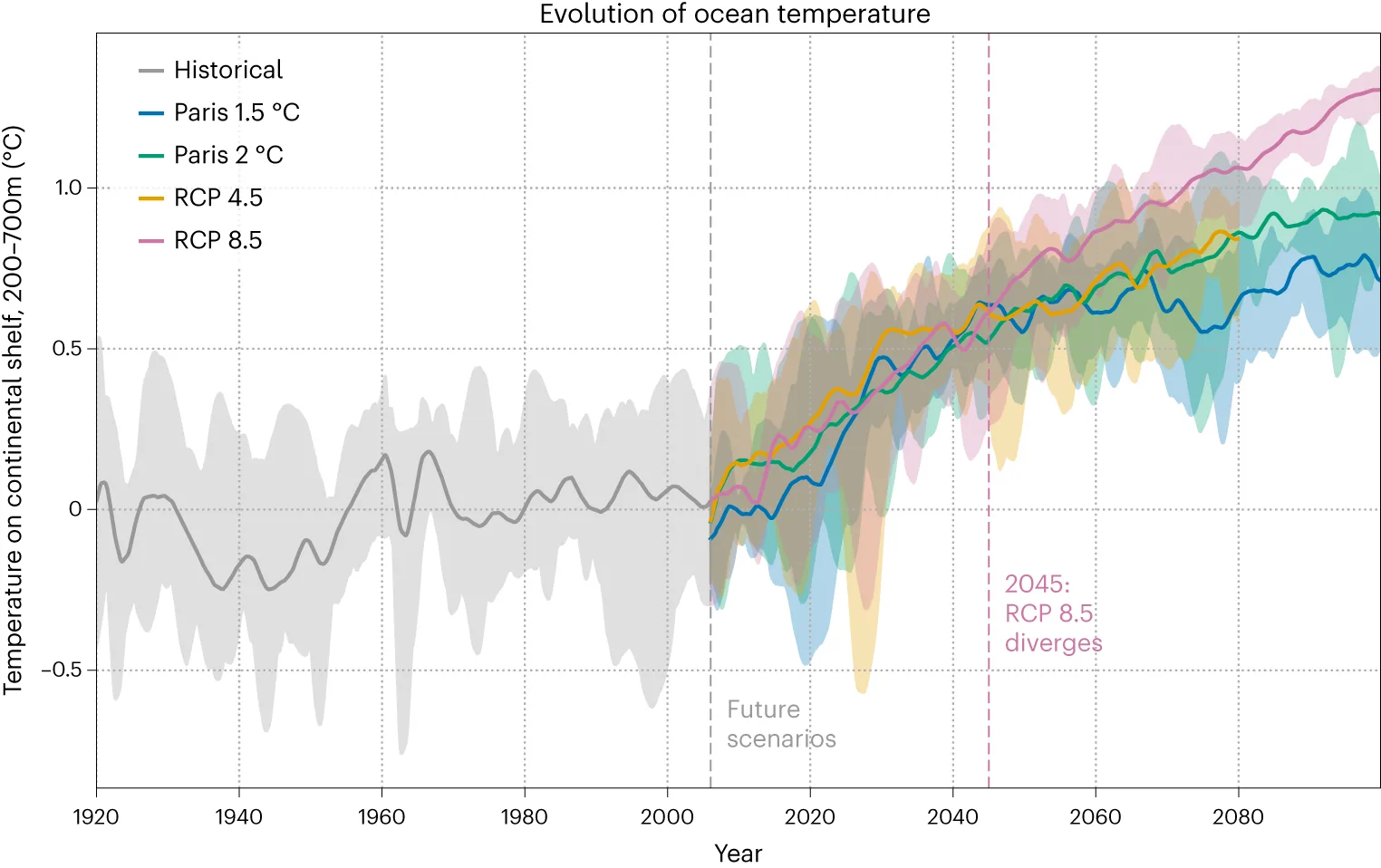
Timeseries of ocean temperature in the core five scenarios. Temperature is averaged over the continental shelf and the depth range 200–700 m, and a 2-yr running mean is applied. For each scenario, the solid line denotes the ensemble mean, while the shaded region shows the full ensemble range. Dashed vertical lines show the onset of future scenarios (2006) and the time at which RCP 8.5 diverges from the other three scenarios (2045).

Although RCP 8.5 has a stronger warming trend than the other future scenarios, this difference is not apparent until mid-century. Timeseries of ocean warming in the core scenarios (Fig. 3) show that all future ensembles are markedly overlapping and have very similar ensemble means for much of the century. RCP 8.5 eventually diverges from the other ensembles, in approximately 2045 (Methods). Therefore, while mitigation of the worst-case climate change scenario still has the potential to reduce Amundsen Sea warming, it will probably not make a difference for several decades. By this time, the impact on some glacier basins of the WAIS could be irreversible, even if ocean temperatures then returned to present-day values.

Scenarios would be expected to diverge more into the twenty-second century and beyond. Although Paris 1.5 °C is not distinct from the two mid-range scenarios when considering trends over the full period, its warming trajectory noticeably flattens out towards the end of the simulation (Fig. 3) and its temperatures diverge in 2059. By this time, the underlying CESM1 scenario requires net negative CO_2_ emissions to stay below 1.5 °C of global warming^15^.

## Mechanism of warming

**Fig. 4:**
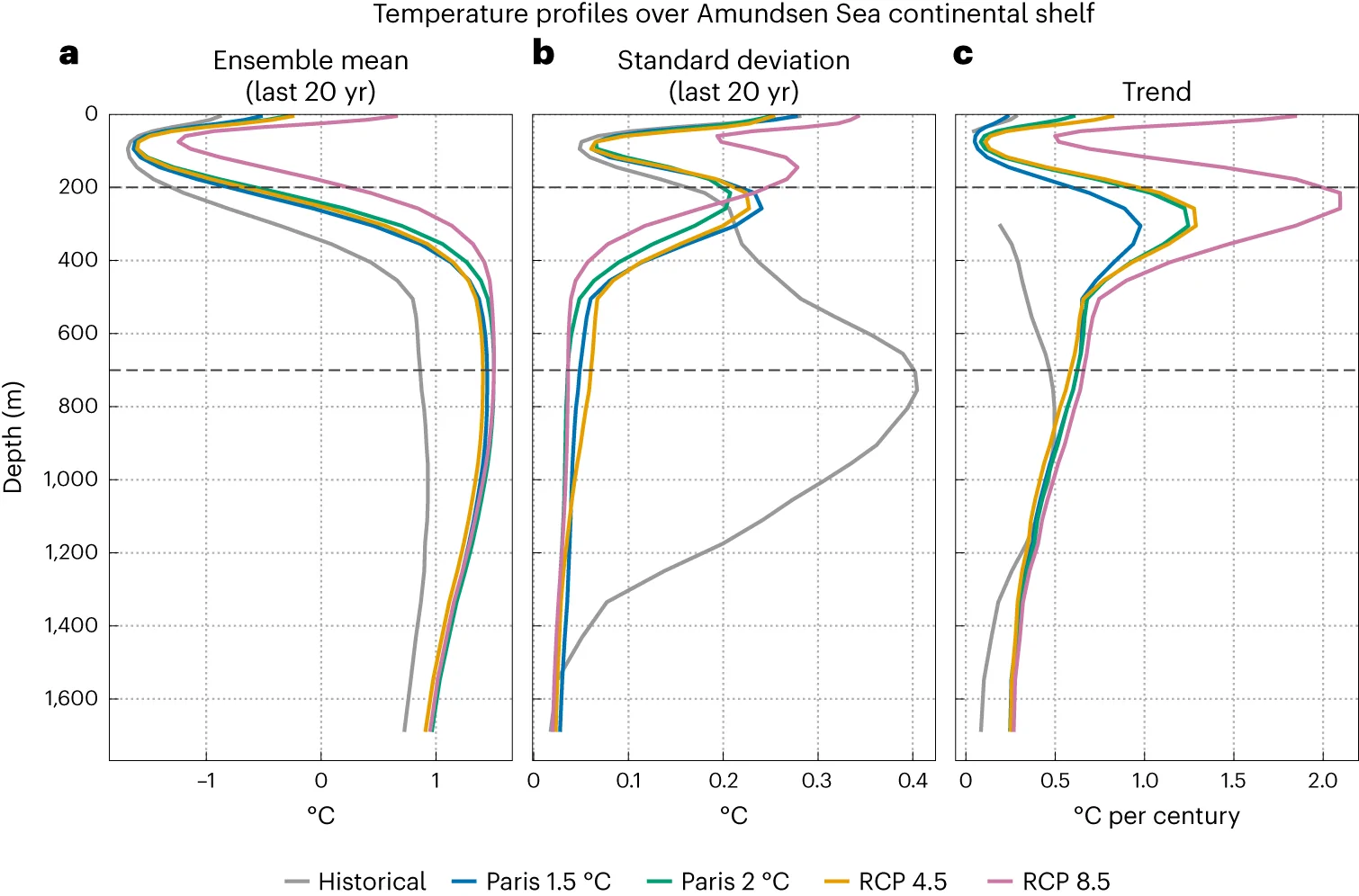
Profiles of ocean temperature in the five core scenarios. Temperature is averaged over the continental shelf. **a**, Ensemble mean temperature over the last 20 yr of the given scenario. **b**, Standard deviation in annual mean temperature over the past 20 yr for all ensemble members. **c**, Ensemble mean trend, calculated using 2-yr running means; lines are not shown for depths where the trend is not significant. Horizontal dashed lines show the depth range 200–700 m used in other analyses.

The oceanographic processes driving warming can be inferred by examining the vertical structure of temperature in the water column. Figure 4 presents temperature profiles averaged over the continental shelf for each core scenario, showing the mean state (Fig. 4a), interannual variability (Fig. 4b) and trends (Fig. 4c). The continental shelf features a seasonal surface layer underlain by a year-round, subsurface, cold Winter Water layer (WW, approx. −1.5 °C) and below that, a warm Circumpolar Deep Water (CDW, ~1 °C). The WW and CDW layers are separated by the thermocline, a sharp temperature gradient around 100–400 m. A peak in standard deviation around this depth (Fig. 4b) indicates interannual variability in the position of the thermocline, which in the present day causes the cavities to oscillate between warm and cold conditions^8,19^.

**Extended Data Fig. 5:**
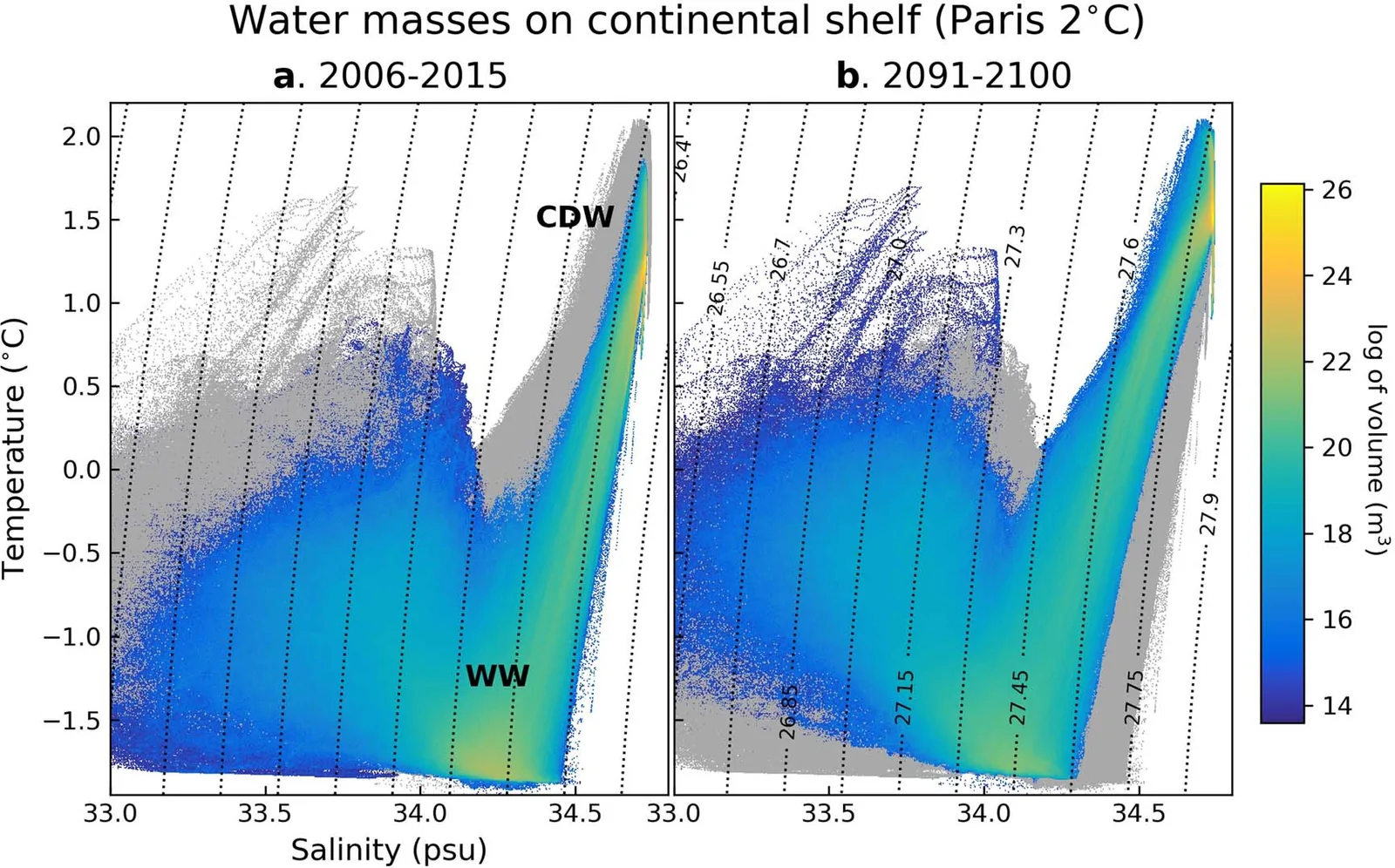
Water mass volumes in temperature-salinity space. The continental shelf region (Fig. 1; all depths) is shown for the Paris 2 °C ensemble. Temperature and salinity are split into 1000 ×1000 bins, and the volume of water in each bin is integrated for each year and ensemble member, using annually averaged values. Shown is the ensemble mean, time mean over **(a)** the first 10 years (2006-2015) and **(b)** the last 10 years (2091–2100). Each panel also shows, in grey, the bins which have zero volume in the given panel but nonzero in the other panel. The log of volume in m3 is plotted for visibility. Black dotted lines show potential density contours in kg/m3-1000. Black labels indicate the water masses WW (Winter Water) and CDW (Circumpolar Deep Water).

**Extended Data Fig. 6:**
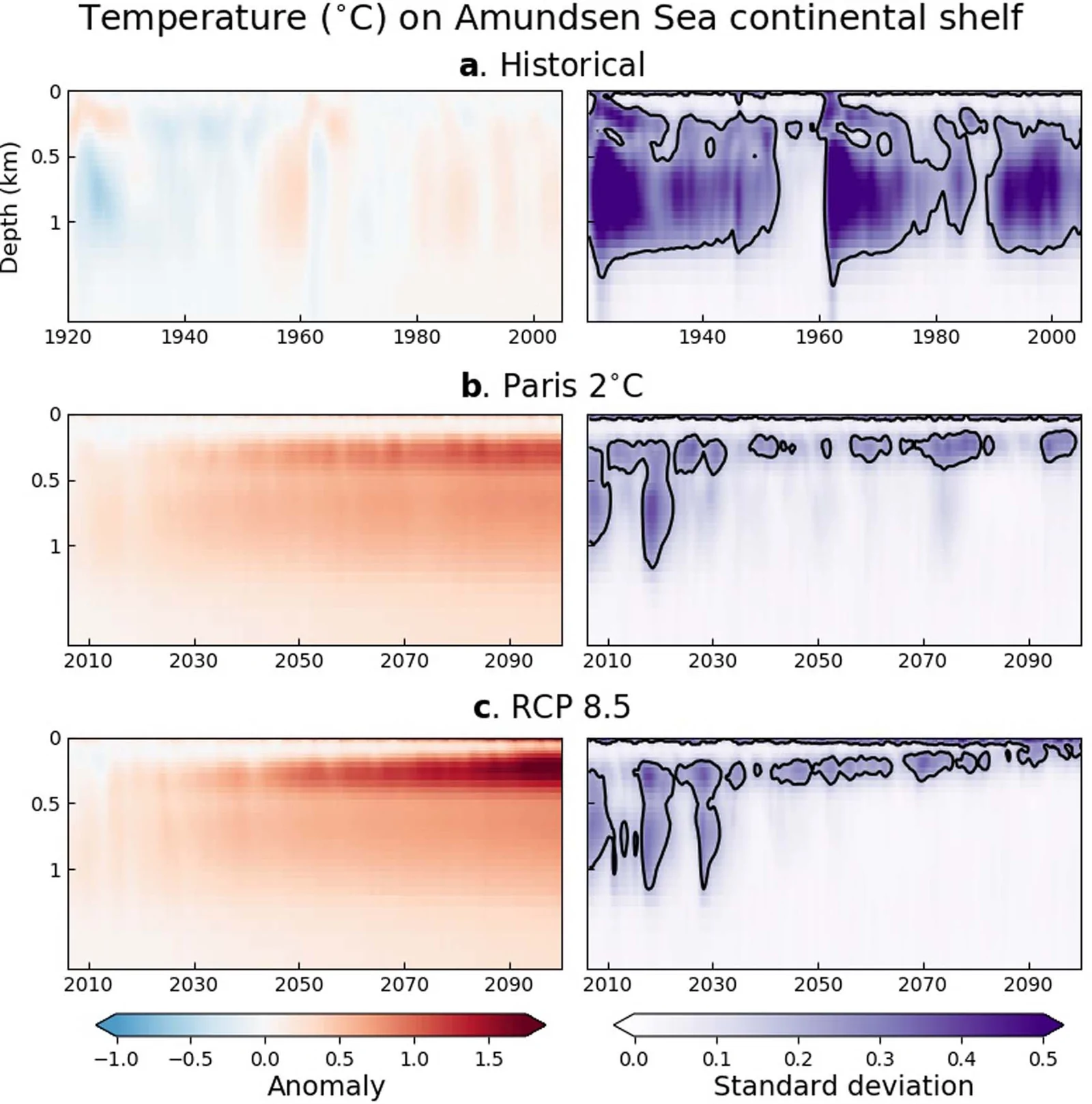
Hovmöller plots (depth versus time) of temperature averaged over the continental shelf region. The left column shows the anomaly from the historical baseline (ensemble mean, time mean over full period 1920–2005). The right column shows the standard deviation across the ensemble, with black contours at 0.2 °C to highlight episodes of convection. Selected scenarios are shown: **(a)** Historical, **(b)** Paris 2 °C, **(c)** RCP 8.5. Data is smoothed using a 12-month running mean.

In all future simulations, the thermocline rises (Fig. 4a) and the warming trend is concentrated at mid-depth (Fig. 4c), indicating a larger volume of warm CDW (Extended Data Fig. 5) which reaches higher up in the water column. In the three lower-forcing scenarios, the peak of the standard deviation (Fig. 4b) remains within the typical depth range of the cavities (200–700 m). Therefore, although cold periods become increasingly less common, there is still some variability in mid-depth temperature. It is only in RCP 8.5 where the thermocline rises so high that its variability no longer strongly affects the cavities. The cavities are continually bathed in warm water and mid-depth temperature trends are markedly stronger than in the other scenarios (Fig. 4c and Extended Data Fig. 6).

In the historical period, a secondary cause of warming is the gradual disappearance of cold convective events on the continental shelf as surface freshening stratifies the water column (Supplementary Discussion 2)^6^. This process has a negligible effect on the future scenarios because convection ceases early in the twenty-first century. Changes in the properties of the CDW core, in the offshore Southern Ocean, are minor (~0.2 °C warming over the twenty-first century). Warming of the continental shelf is therefore driven by an increased volume of CDW, rather than increased temperature of the water mass itself.

**Fig. 5:**
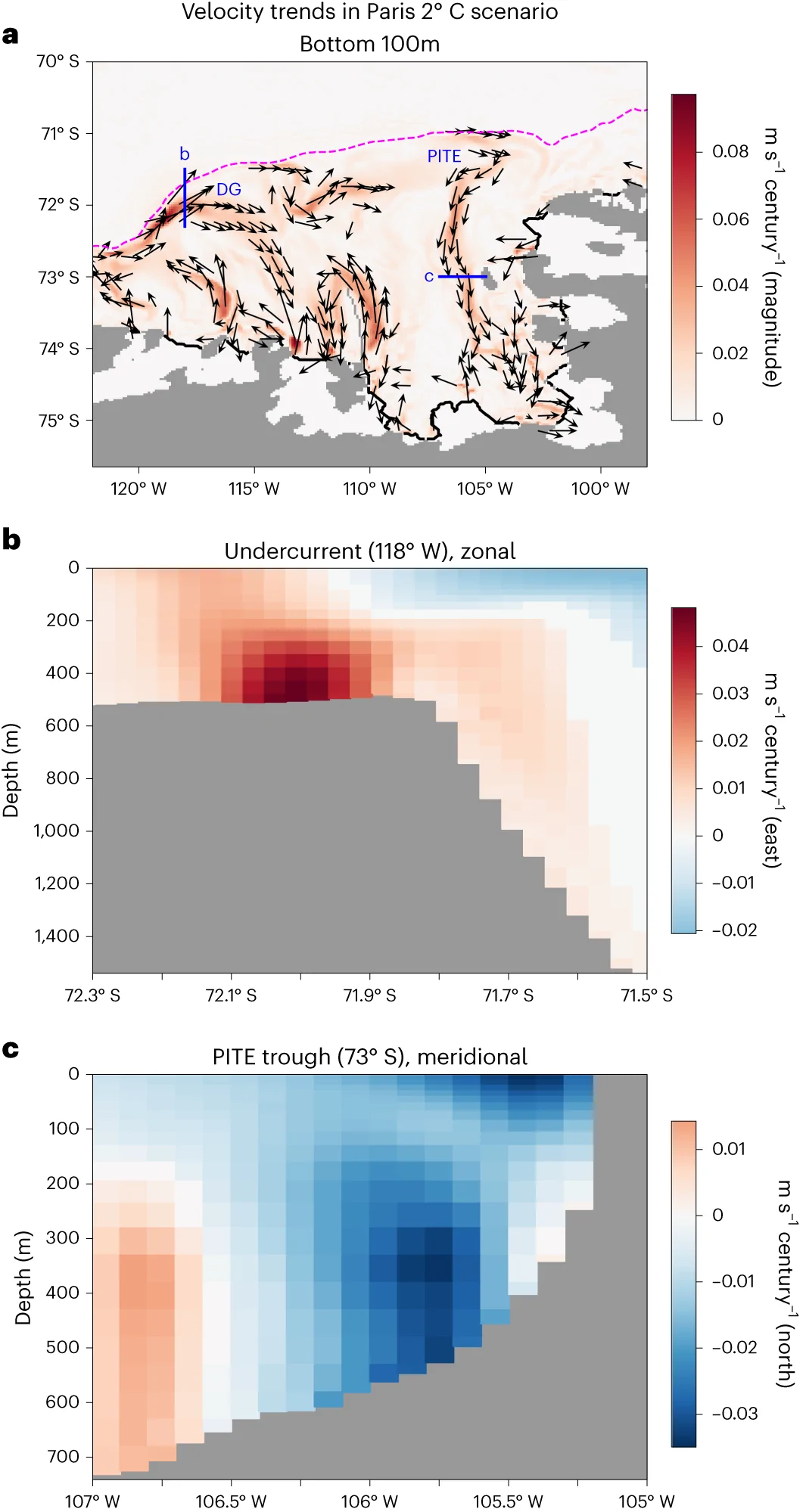
Ensemble mean trends in velocity for the Paris 2 °C scenario. Trends are computed using annual averages. **a**, Trends in the bottom 100 m, zoomed into the continental shelf. The magnitude of the trends is shaded in red, and vectors are plotted where the magnitude exceeds 0.02 m s^−1^ century^−1^. Blue lines show the transects used for **b** and **c**. Blue labels indicate the PITE and DG Troughs. The dashed magenta line is the 1,000 m isobath of the continental shelf break. **b**, Trends in zonal velocity (red, eastward trend; blue, westward) across the undercurrent transect at 118° W. **c**, Trends in meridional velocity (red, northward trend; blue, southward) across the PITE transect at 73° S. Where trends are not significant, the colour is set to white and/or the given vector component(s) set to zero.

Rising of the thermocline is driven by intensified circulation over the continental shelf and slope, bringing a larger volume of CDW onshore. The Amundsen Undercurrent, a bottom current transporting CDW eastward along the shelf break^20^, strengthens in our simulations (Fig. 5a,b). Further downstream, the undercurrent turns onshore and transports CDW southward through bathymetric troughs (Extended Data Fig. 1a). This simulated onshore transport also strengthens, particularly in the Pine Island Thwaites East Trough (PITE; Fig. 5a,c) as well as in the Dotson-Getz Trough (Fig. 5a).

**Extended Data Fig. 7:**
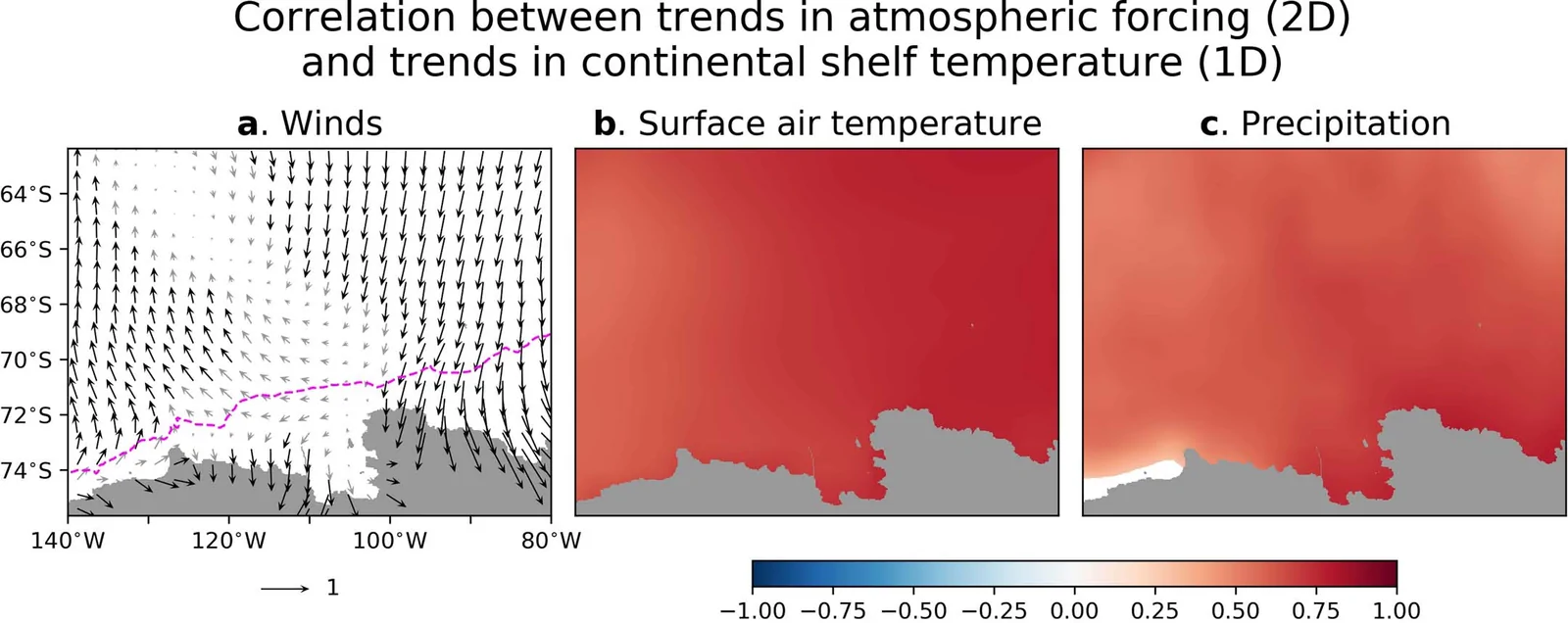
Maps of correlation between trends in atmospheric forcing variables and trends in continental shelf ocean temperature. Temperature is averaged over 200-700 m and the region shown in Fig. 1. Inter-ensemble correlations are calculated using all members of the five core scenarios, and the individual trends are calculated using annual averages over the full duration of each simulation. Correlations are shown for: **(a)** Surface winds, with black vectors where at least one component (zonal or meridional) is significant at the 95% level. Grey vectors indicate neither component is significant. The dashed magenta line is the 1750m isobath of the continental shelf break. **(b)** Surface air temperature and **(c)** precipitation, only plotted where the correlation is significant at the 95% level. Atmospheric forcing variables which are closely related to surface air temperature and/or less important to the ocean (surface humidity, surface pressure, shortwave and longwave radiation) are not shown.

The increased CDW flux warms the continental shelf and the adjacent ice-shelf cavities. Individual trends in continental shelf temperature significantly correlate with trends in southward transport through the PITE Trough (Extended Data Table 2, except for the intra-ensemble correlation for Paris 2 °C). This relationship, combined with existing understanding of Amundsen Sea circulation from both observations and models^21,22^, indicates that undercurrent intensification is the primary driver of Amundsen Sea warming and ice-shelf melting in our simulations. The atmospheric drivers of these oceanographic changes are less conclusive, but appear to be linked to surface warming and increased precipitation (Supplementary Discussion 3 and Extended Data Fig. 7), rather than winds as proposed by previous work^6,9^. However, no dominant driver that can explain the variability in trends within each ensemble has been identified.

## Relevance to sea-level rise

Increased ice-shelf basal melting can result in a loss of buttressing, increased mass flux across the grounding line and ultimately sea-level rise. Because our ocean simulations are not coupled to an ice-sheet model, we cannot quantify the sea-level rise contribution implied by our findings. However, we can indirectly assess their importance for sea-level rise on the basis of the spatial distribution of the basal melting trends. Buttressing provided by ice-shelves is heterogeneous: increased basal melting in crucial regions triggers a disproportionate loss of grounded ice, while the same increase in melting elsewhere may have little impact.

**Fig. 6:**
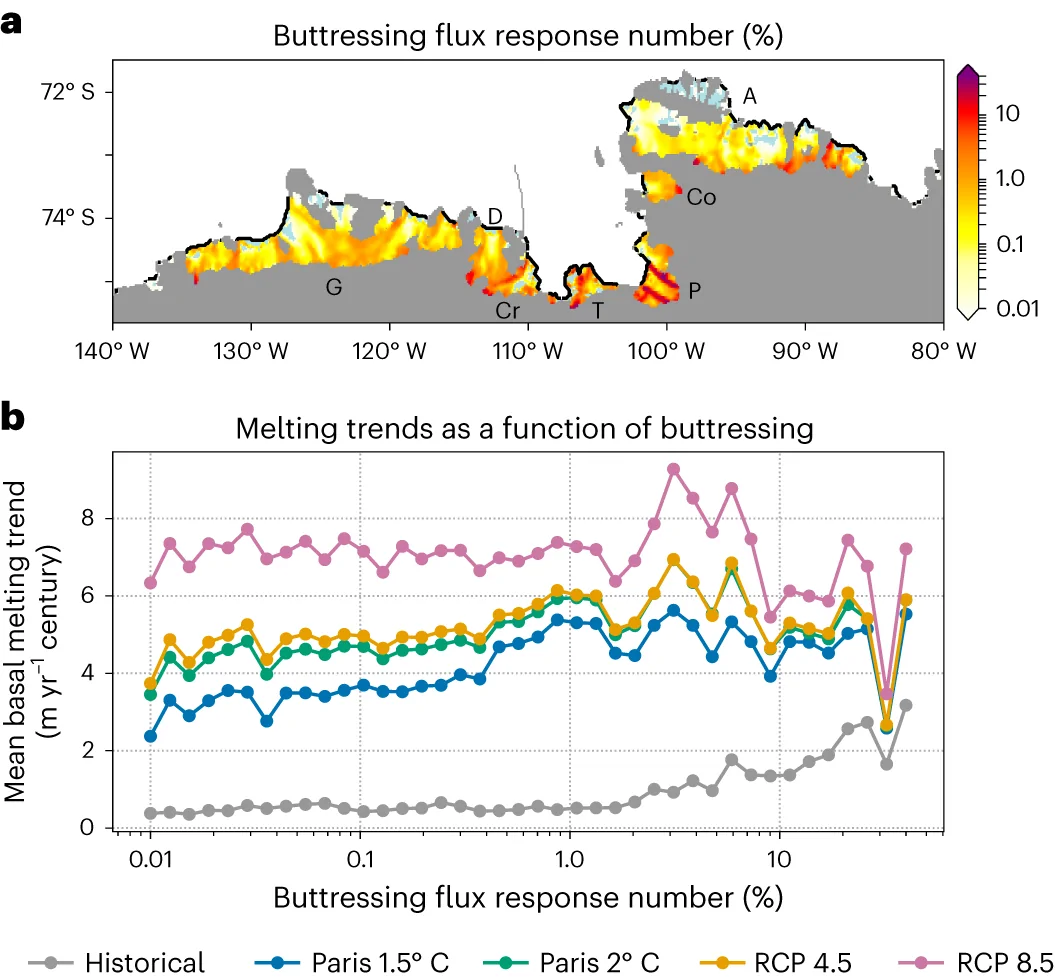
Implications of simulated melting for ice-shelf buttressing. **a**, Effect of local ice-shelf thinning on sea-level rise, calculated for the Amundsen Sea using a standalone ice-sheet model (Methods). The BFRN, following ref. ^23^, is the ratio of total changes in mass flux across all grounding lines to the magnitude of locally applied ice-shelf thinning. Higher values (note log scale) indicate ice-shelf thinning in the given region causing more sea-level rise. Negative values, indicating ice-shelf thinning that reduces grounding line flux, are masked in light blue. Black labels indicate ice shelves as in Fig. 1. **b**, Ensemble mean ice-shelf basal melting trends as a function of BFRN for each core scenario. The BFRN values in **a** are split into 40 bins, following a log scale. All values <0.01%, including negative values, are contained within the first bin. Melting trends are averaged over the regions corresponding to each bin; where the ensemble mean trends are not significant, they are set to zero.

Here we replicate the methodology of ref. ^23^ to calculate the buttressing flux response number (BFRN), a spatially varying metric that quantifies ice-shelf buttressing. Regions with high BFRN have greater potential to cause sea-level rise if they experience increased basal melting. We use the Úa ice-flow model (Methods) to calculate BFRN across every ice shelf in our domain, in much greater detail than was available previously (Fig. 6a). The regions with highest BFRN include the grounding lines of most ice shelves, the shear margins of Pine Island and the shear margin bisecting the Thwaites ice shelf.

**Extended Data Fig. 8:**
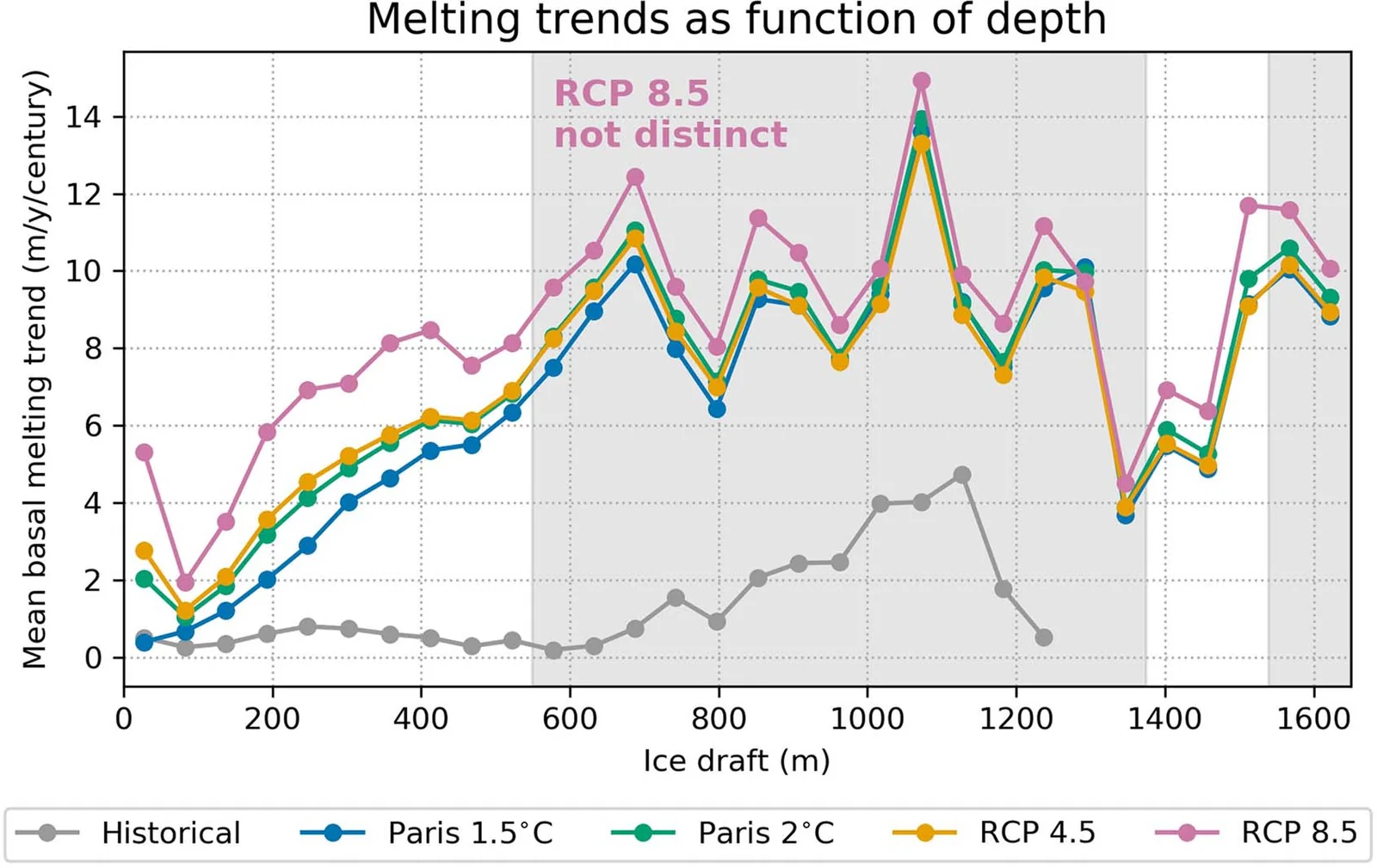
Ice-shelf basal melting trends as a function of depth of the ice draft. Conventions follow Fig. 6b; here 30 bins are defined following a linear scale. Missing points indicate that the given scenario has no significant trends in the given bin. Grey shading indicates that, when considering ensemble spread (not shown), RCP 8.5 is not distinct from the other future scenarios for the given depth class (Methods).

Using the BFRN field to define ice-shelf classes, we express ice-shelf melting trends as a function of buttressing (Fig. 6b). The future scenarios show increased melting of all classes, including the most glaciologically important classes (BFRN > 10%). This result also holds if buttressing classes are instead defined by depth of the ice draft (Extended Data Fig. 8). The curves in Fig. 6b are relatively flat, indicating that future projections of melting do not disproportionately affect either low or high BFRN regions; conversely, the historical trends are concentrated in classes with high BFRN.

Comparing the different future scenarios, our main findings from ocean warming also hold for buttressing implications: the three lower-range scenarios are very similar, while RCP 8.5 has a higher ensemble mean trend. The differences are largest for the lower values of BFRN, and when considering ice draft depth classes rather than BFRN, the differences are not significant for most of the deeper ice (Extended Data Fig. 8). That is, additional increases in melting in RCP 8.5 are disproportionately among ice-shelf classes that have less potential to cause sea-level rise. This occurs because the additional thermocline rise in RCP 8.5 (Fig. 4) primarily affects shallower ice drafts, which tend to have lower BFRN. Deeper ice, with generally higher BFRN, becomes engulfed by CDW in all future scenarios. Even mitigation of the worst-case scenario therefore may not substantially reduce the future sea-level rise contribution from this sector of the WAIS.

## Implications

Our simulations present a sobering outlook for the Amundsen Sea. Substantial ocean warming and ice-shelf melting is projected in all future climate scenarios, including those considered to be unrealistically ambitious. A baseline of rapid twenty-first-century ocean warming and consequent sea-level rise appears to be committed. This warming is primarily driven by an acceleration of the Amundsen Undercurrent transporting warmer CDW onto the continental shelf. Basal melting increases across all ice shelves in the Amundsen Sea, including in regions providing critical buttressing to the grounded ice sheet.

Mid-range mitigation scenarios (RCP 4.5) and the more ambitious aims of the Paris Agreement (global warming limited to 1.5 °C or 2 °C) are statistically indistinguishable in terms of Amundsen Sea warming trends over the twenty-first century. The similarity of ocean warming between forcing scenarios and the large spread within each ensemble imply that internal climate variability will be extremely important in determining the future of the WAIS. The only control that mitigation can offer is by preventing the worst-case scenario (RCP 8.5). Here, the thermocline rises so high that most ice shelves are permanently bathed in warm CDW. However, RCP 8.5 does not diverge from the lower-range scenarios until 2045, and the additional melting mainly affects shallower regions of the ice shelves, which are less important for sea-level rise. The choice of scenario is likely to become more important in the twenty-second century and beyond.

This study presents, to our knowledge, the most comprehensive future projections of Amundsen Sea ice-shelf melting so far. We simulate a wide range of future climate scenarios, and by running ensembles we can compare these scenarios in a statistically robust manner. Ensembles also allow us to study the distribution of possible melting trends, considering low-probability, high-impact cases at the upper end of the distribution, as well as the most likely case. By combining the maximum future warming trend in our ensembles (Fig. 2) with historical warming, we find that Amundsen Sea ocean conditions in 2100 could be up to 2 °C warmer than pre-industrial temperatures. For Antarctic water masses, a 2 °C increase is striking.

Although this study is a major advance, further research and model development are needed to increase confidence in our conclusions. Our study uses a single ice–ocean model forced by a single climate model and does not consider feedbacks related to ice-shelf geometry (Supplementary Discussion 4). Furthermore, our future projections focus on oceanic forcing of the WAIS, which is currently the dominant process driving mass loss^2^. In the longer term, atmospheric forcing may become increasingly important, as it affects the surface mass balance of the ice sheet. In strong forcing scenarios beyond 2100, increased surface melting could trigger ice-shelf collapse in the Amundsen sector and rapidly accelerate mass loss^24–27^. Conversely, increased snowfall in a warmer climate could offset sea-level rise^11^. We do not consider these processes in our study, but they could introduce a stronger sensitivity to the climate forcing scenario. Finally, the long timescales of ice-sheet dynamics mean that the WAIS could continue to lose mass even if ocean temperatures do not increase further^28,29^. However, continued ocean warming will accelerate the rate of mass loss and will trigger more impacts of sea-level rise on timescales which are immediately policy-relevant.

This study does not undermine the importance of mitigation in limiting the impacts of climate change. Mass loss from the WAIS is just one component of sea-level rise, and other regions of Antarctica are unlikely to lose substantial mass if current emissions targets are met^30^. This is to say nothing of the many impacts of climate change beyond sea-level rise. However, adaptation should now be considered more seriously as a priority in the world’s response to sea-level rise. The opportunity to preserve the WAIS in its present-day state has probably passed, and policymakers should be prepared for several metres of sea-level rise over the coming centuries. Internal climate variability, which we cannot predict or control, may be the deciding factor in the rate of ice loss during this time. Limiting the societal and economic costs of sea-level rise will require a combination of mitigation, adaptation and luck.

## Methods

### Ice–ocean simulations

Simulations were performed using the previously described configuration of MITgcm^6^. This model covers the Amundsen Sea region at tenth-degree resolution (~3–5 km depending on latitude) and includes components for the ocean, sea-ice, and ice-shelf thermodynamics^31,32^. The bathymetry, ice draft and grounded ice mask are held fixed at present-day conditions, following MEaSUREs BedMachine v.2 (refs. ^5,33^). All parameters and parameterizations are as in ref. ^6^, which also validated the model’s performance during the observational period. The model generally agrees with the observational record of continental shelf temperature and salinity, in terms of mean conditions as well as interannual variability. The Amundsen Undercurrent in this configuration was also previously assessed^22^ and confirmed to be the main driver of variability in onshore heat content and ice-shelf basal melting.

The simulations presented here are forced by a suite of climate model experiments using CESM1, as detailed in Extended Data Table 1. The atmospheric forcing is bias corrected using the same spatially varying, time-constant correction fields used in ref. ^6^. New to this study is the use of transient boundary conditions on the lateral ocean boundaries (140° W, 80° W, 62° S) using bias-corrected CESM1 forcing in all of the main ensembles; the bias-correction methodology is described in the next section. CESM1 was chosen for its availability of large ensembles for multiple scenarios, as well as its good representation of winds over the Amundsen Sea^6,9,10^. These winds are important drivers of the circulation features within our domain, including the Amundsen Undercurrent as well as the southern branch of the Antarctic Circumpolar Current.

We used a subset of the available ensemble members in CESM1, balancing statistical robustness with computational cost and data availability. The core Historical experiment (10 members) followed historical external forcing from 1920–2005^14^, considering both anthropogenic (greenhouse gases, ozone depletion, aerosols, land use change) and natural (solar, volcanic) sources. To isolate the impact of transient boundary conditions, Historical Fixed BCs (5 members) instead forced the ocean boundaries with a repeating present-day monthly climatology from the World Ocean Atlas 2018^34^ and the Southern Ocean State Estimate^35^. Both historical scenarios were initialized as in ref. ^6^ and spun up for 30 yr by repeating the period 1920–1949 once.

Four core future scenarios following different forcing pathways (Paris 1.5 °C, Paris 2 °C, RCP 4.5, RCP 8.5) branched from the corresponding members of the Historical scenarios^14–16^. Ten members were used for all scenarios except for Paris 1.5 °C (5 members), for which missing data precluded the use of members 6–10. One additional scenario with climatological boundary conditions, RCP 8.5 Fixed BCs (5 members), branched from the end of Historical Fixed BCs.

### Bias-correction methodology at open boundaries

CESM1 is a global climate model with relatively coarse resolution (~1°) and is not optimized for the Antarctic continental shelf. Consequently, it exhibits biases in Antarctic water masses, including a surface fresh bias and an overly shallow thermocline, which would be imported into our regional model if the raw CESM1 output fields were used to force MITgcm at the open ocean boundaries. Amundsen Sea circulation and ice-shelf melting are very sensitive to the underlying water mass structure^36,37^ and such biases would negatively impact MITgcm’s present-day realism and undermine confidence in its future projections. Therefore, we have developed a bias-correction methodology that preserves transient changes in CESM1’s water mass properties (for example, warming) and structure (for example, thermocline shoaling), without inheriting mean-state biases in either characteristic.

Key to our methodology is the concept of ‘normalized T/S space’, in which water masses are indexed on the basis of their relative temperature and salinity. Each axis ranges from 0 (the coldest or freshest water on the given open boundary) to 1 (the warmest or saltiest), with a linear transformation. Extended Data Fig. 2 shows an example water mass distribution in normalized T/S space for the CESM1 present-day climatology (Extended Data Fig. 2a) and the World Ocean Atlas (WOA, Extended Data Fig. 2b). The transformation is calculated separately for the two products, so their distributions are broadly similar even though the absolute values are different (Extended Data Fig. 3a,b).

For each CESM1 scenario, ensemble member, month and model boundary, we bias-corrected ocean temperature and salinity in 5 main steps:

1. **Transformed to normalized T/S space**. We calculated this transformation separately for the CESM1 (Extended Data Fig. 2a) and the WOA (Extended Data Fig. 2b) climatologies. The normalized T/S space was split into 100 × 100 bins. We also calculated the CESM1 temperature and salinity anomalies, with respect to climatology, for each bin (Extended Data Fig. 2c,d).
2. **Filled in the gaps to define temperature and salinity anomalies in every possible bin**. Each missing bin (zero volume in CESM1) was filled with the weighted mean of its 10 nearest valid neighbours, where the weights are the inverse of the Cartesian distance between indices, such that closer neighbours are weighted more heavily. The surface was then smoothed using a Gaussian filter with a standard deviation of 2 bins (Extended Data Fig. 2e,f).
3. **For every bin with non-zero volume in WOA, we looked up the temperature and salinity anomalies**. We added these anomalies to the WOA climatology. The bias-corrected values were therefore (CESM1 time-varying) − (CESM1 climatology) + (WOA climatology), but with a transformation to normalized T/S space after the first two terms.
4. **Transformed the corrected values back to physical space, using the WOA water mass distribution**. The correct anomalies had now been applied to the correct water masses, even if their initial properties were biased and even if they were in the wrong position in the water column. For example, the warmest and saltiest water in WOA was assigned the same anomaly as the warmest and saltiest water in CESM.
5. **Special treatment of the mixed layer**. For consistency, sea surface temperature and salinity anomalies should agree with the surface fluxes implied by the atmospheric forcing. Special treatment of the mixed layer was required to avoid occasional conflicts where the surface water in WOA wrongly inherited the subsurface anomalies from CESM1, as these two regions were very close on the T/S distribution and could have opposite anomalies (for example, surface freshening and subsurface salinification as the thermocline rises: Extended Data Figs. 2f and 3e). To avoid these issues, we selected the CESM1 sea surface temperature and salinity anomalies in physical space, with no transformation to normalized T/S space. Over the depth of the mixed layer, we linearly transitioned from the surface anomalies to the original anomalies calculated in steps 1–4. We selected the mixed layer base in the WOA climatology using a potential density threshold of 0.1 kg m^−3^ above the surface density at each point. Finally, a minimum temperature of −1.9 °C (surface freezing point) was enforced at all depths.

The overall bias-correction methodology has been tested extensively and adequately preserves trends in CESM1 water mass properties and structure, while also preserving the more realistic water mass structure of the World Ocean Atlas. An example of its effectiveness is shown in Extended Data Fig. 3 for salinity on the eastern boundary. Compared with WOA (Extended Data Fig. 3a), the CESM1 climatology (Extended Data Fig. 3b) has a fresh bias in the surface and mixed layer, and the halocline (equivalent to the thermocline) is too shallow. By 2100 (Extended Data Fig. 3c), CESM1 has an even fresher surface and mixed layer, and an even shallower thermocline. The bias-correction methodology preserves these changes while removing the initial biases in both water mass properties and structure, creating a final salinity field that looks realistic (Extended Data Fig. 3d). Comparing the raw anomalies in CESM1 (Extended Data Fig. 3e) to the corrected anomalies that are ultimately applied to MITgcm (Extended Data Fig. 3f) shows that the magnitude and character of CESM1’s anomalies have been preserved. Both panels show a similar freshening of the mixed layer and salinification of the subsurface as the thermocline rises. However, the corrected anomalies have a different vertical structure, following the deeper thermocline of the World Ocean Atlas.

We took a simpler approach to velocity and sea-ice variables on the boundaries, which were linearly bias corrected in physical space. That is, the CESM1 anomaly field was added to the monthly climatology from the Southern Ocean State Estimate, with no transformation in between. MITgcm was not particularly sensitive to transient changes in these variables at the lateral boundaries; indeed, momentum anomalies do not persist within the domain to the extent that tracers do, and sea-ice variables quickly adjust to the surface fluxes.

### Statistical conventions

To reduce calculations to a single dimension, we analysed trends in temperature averaged over the continental shelf region in Fig. 1 and the depth range 200–700 m. These bounds approximated the typical ice draft and seafloor depth at the ice front (Extended Data Fig. 1), therefore spanning the typical depth range at which the cavities are open to the surrounding ocean. Trends in ice-shelf melting were calculated using the basal mass loss integrated over ice shelves between Dotson and Cosgrove inclusive, which are the cavities directly affected by the corresponding ocean region. All statistical calculations used the 95% significance threshold.

The significance of an ensemble mean trend was determined by a 2-sided *t*-test across the *n* individual trends, where *n* is the ensemble size, given the null hypothesis that the mean is zero. To determine whether trends between two ensembles are distinct, we applied a 2-sided, 2-sample *t*-test with unequal sample size and variance. This test is appropriate because the matching ensemble members in different scenarios are not paired^14^. The correlation between trends in two variables (Extended Data Table 2) was calculated using a linear regression across the given ensemble(s).

Note that RCP 4.5 ends in 2080, due to unavailability of CESM1 forcing, while the other future scenarios end in 2100. All statistical comparisons of trends (such as determining which scenarios are distinct, or inter-ensemble correlations) were calculated over the period of overlap, that is, 2006–2080 if RCP 4.5 is included and 2006–2100 otherwise. Figures and tables show trends calculated over the full simulation period, including to 2100 where available. When the warming and melting trends were recalculated to all end in 2080 (Extended Data Table 1), they increased slightly, particularly in the two Paris scenarios. This is because greenhouse gas emissions level off towards the end of the century. Our main conclusions were not affected by the choice of end date for the trends.

To calculate the year of divergence of a given scenario (for example, RCP 8.5) from the others, we compared the 10 ensemble members of RCP 8.5 to the 25 ensemble members of the other three scenarios combined. For each member, we calculated annual means in ocean temperature (200–700 m mean, averaged over the continental shelf region). For each year, we considered the 11-yr window centred on the given year to account for interannual variability. This produced two samples to compare for each year: a sample of 110 values from RCP 8.5 (10 members × 11 yr) and a sample of 275 values from the other scenarios combined (25 members × 11 yr). To determine whether the two samples were distinct, we performed a 2-sample *t*-test as before. The year of divergence is the year at which the samples become distinct and remain distinct for the remainder of the simulation.

A similar method was used to determine the values of the BFRN (Fig. 6b) or ice-draft depth class (Extended Data Fig. 8) for which RCP 8.5 was distinct from the other scenarios. For each bin, the two samples were compared as above, but without using a window. That is, the samples were composed of 10 values for RCP 8.5 and 25 values for the other scenarios, corresponding to the individual ensemble members for that bin only.

### Ice-shelf buttressing simulations

To quantify the effect of ice-shelf thinning on the mass flux across the grounding line, we calculated the buttressing flux response numbers *θ*_B_ following the methodology of ref. ^23^. We used a regional configuration of the ice-sheet model Úa^38^, which includes the same ice shelves as in the MITgcm and their corresponding drainage basins. The model domain was bounded by a fixed ice front (as in Fig. 1) and the ice divide of the H-Hp, G-H and F-G drainage basins^39^. Úa treats the ice as a non-Newtonian viscous fluid with Glen’s flow law exponent *n* = 3 and a shallow-shelf approximation of the momentum equations. Basal sliding was represented by a nonlinear Weertman-type sliding law with exponent *m* = 3. A spatial distribution of the rate factor in Glen’s law and slipperiness in the sliding law were obtained through a commonly used optimization procedure^40^. Bedrock geometry and ice thickness were linearly interpolated from the BedMachine v.2 dataset^5,33^, as used for the ocean domain, and surface velocities were taken from the MEaSUREs dataset^41^. A zero-flow condition was imposed for grounded ice at the model boundary. All calculations in Úa were performed using finite-element methods on an unstructured grid. Linear elements were used, with a nominal nodal spacing of 2.5 km, local mesh refinement of 1 km in areas with high effective strain rates and strain rate gradients, and 750 m in the vicinity of the grounding line.

To calculate the buttressing flux response numbers, floating areas in the model domain were divided into 5 × 5 km squares. For each square, the thickness of floating ice within the square was reduced by 1 m, and a diagnostic model calculation was performed to estimate the resulting change in ice velocity. This allowed us to calculate a spatial distribution of *θ*_B_ at 5 km resolution, following ref. ^23^:

$${\theta }_{\rm{B}}=\frac{{\Delta q}_{{\rm{GL}}}}{\int \rho \Delta {hdxdy}}$$

where Δ*q*_GL_ is the difference in annual grounding line flux (in kg) between the perturbed and unperturbed geometry, *ρ* = 917 kg m^−3^ is the ice density and Δ*h* (in m) is the difference between the perturbed and unperturbed ice-shelf thickness. In the calculation of Δ*q*_GL_, changes in flow across the grounding line of ice rises, ice rumples and islands were omitted, and only the flux across the main grounding line separating the ice shelves and the continent was considered. The 5-km resolution of our calculation, as compared with 20 km in ref. ^23^, was enabled by the high mesh resolution of our regional model setup. Moreover, the resolution of *θ*_B_ was comparable to that of the ocean model, which allowed for a more robust comparison between *θ*_B_ and trends in melt rate. It also allowed us to resolve finer spatial variability in *θ*_B_ for the small and geometrically complex ice shelves within the domain, underscoring the important role of shear margins and ice near the grounding line for ice-shelf buttressing.

## Online content

Any methods, additional references, Nature Portfolio reporting summaries, source data, extended data, supplementary information, acknowledgements, peer review information; details of author contributions and competing interests; and statements of data and code availability are available at https://doi.org/10.1038/s41558-023-01818-x.

## Supplementary information

## Figures and Tables

**Extended Data Table 1:**
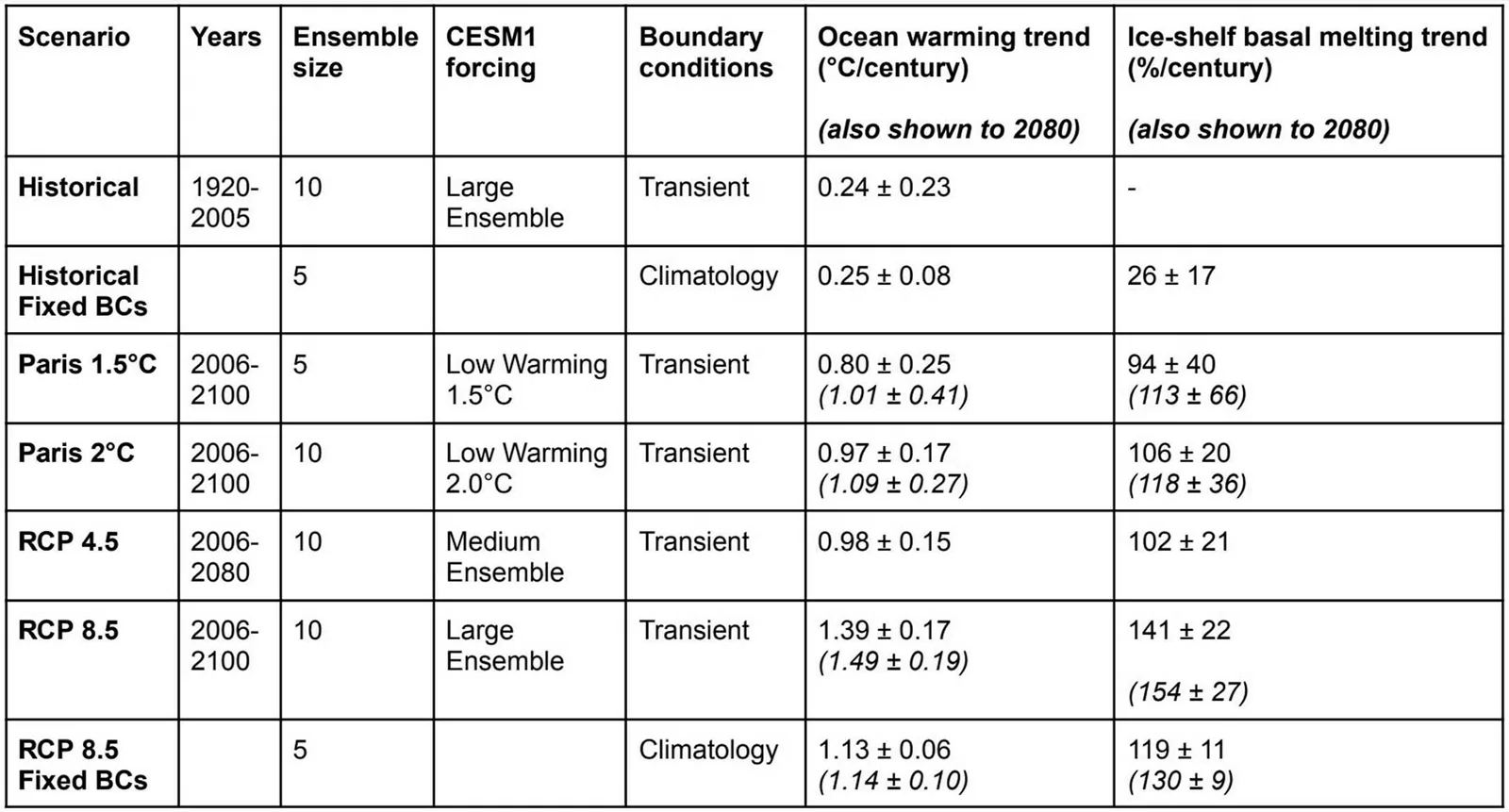
Details of MITgcm simulations analysed in this paper

‘Scenario’ denotes the name used in the text. ‘Years’ shows the time span of each scenario (inclusive). ‘Ensemble size’ shows the number of ensemble members; 10 indicates that the corresponding CESM1 members 1-10 were used for forcing, for example. ‘CESM1 forcing’ indicates the Community Earth System Model experiment used for forcing of atmospheric variables and (if applicable) lateral ocean boundaries. ‘Boundary conditions’ indicates whether the lateral ocean boundaries are forced by a present-day climatology derived from observations (‘Climatology’) or by transient CESM1 fields bias corrected to the same observational products (‘Transient’). ‘Ocean warming trend’ and ‘Ice-shelf basal melting trend’ show the ensemble mean trend, plus or minus one standard deviation, of ocean temperature (averaged over the continental shelf region in Fig. 1 and from 200-700 m depth) and ice-shelf basal melting (integrated over ice-shelves between Dotson and Cosgrove inclusive, and expressed as a percentage of the ensemble mean over the first 30 years of the Historical experiment), calculated using 2-year running means. Trends which are not significant at the 95% level are denoted by ‘-’. Trends are calculated over the duration of the given experiment; for future scenarios which end in 2100, the trend ending in 2080 is also reported (italicized, in parentheses) for direct comparison to RCP 4.5. Blank boxes indicate that the information is the same as the above experiment.

**Extended Data Table 2:**
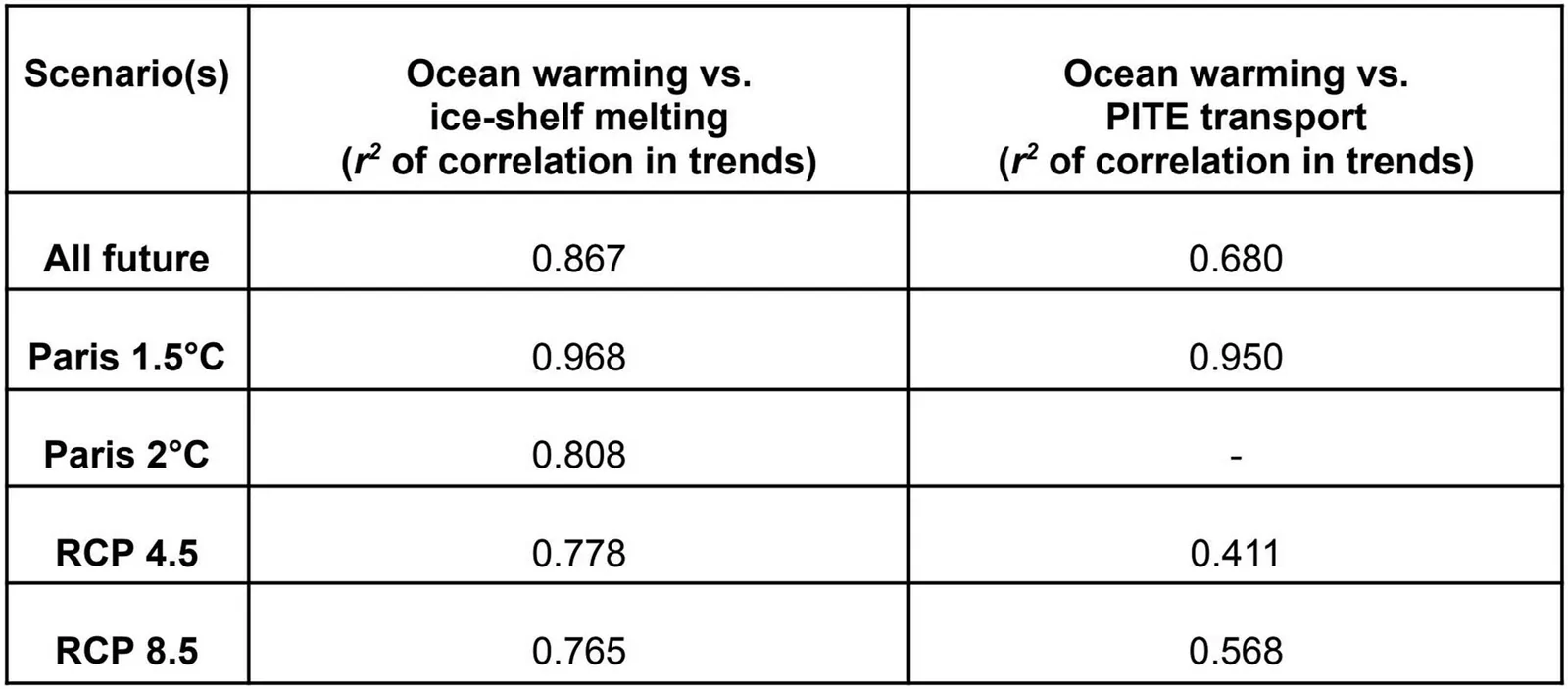
Coefficient of determination (r^2^) for the member-wise correlation in trends between the two given variables

We calculate the inter-ensemble correlation, considering trends in all ensemble members of all four future scenarios (‘All future’), as well as the intra-ensemble correlations for each scenario individually. ‘Ocean warming’ refers to temperature averaged over the continental shelf region in Fig. 1 and between 200-700 m, ‘ice-shelf melting’ to ice-shelf basal mass loss integrated over ice-shelves between Dotson and Cosgrove inclusive, and ‘PITE transport’ to southward transport integrated over the transect shown in Fig. 5c. Correlations which are not significant at the 95% level are denoted by ‘-’. Trends are calculated over 2006-2080 if RCP 4.5 is included, and 2006-2100 otherwise.

## Data Availability

A processed version of the model output is publicly accessible at the UK Polar Data Centre^42^. Due to its large size, the full unprocessed dataset is not hosted on a public-facing server, but subsets can be transferred on an individual basis as required. To obtain model output beyond the publicly accessible version, please contact the corresponding author.

## Code availability

The ocean model source code and configuration files are publicly accessible^43^, as is the pre- and post-processing code^44^ and the ice sheet model source code^38^.
