# Supplementary material for: Unavoidable future increase in West Antarctic ice-shelf melting over the twenty-first century
Source: Nat Clim Chang. 2023 Oct 23;13(11):1222–8. doi: 10.1038/s41558-023-01818-x (PMC13538064; doi:10.1038/s41558-023-01818-x)
Supplement: Supplementary file 1 [file 41558_2023_1818_MOESM1_ESM.pdf]

nature climate change

Article

https://doi.org/10.1038/s41558-023-01818-x

Unavoidable future increase in West Antarctic ice-shelf melting over the twenty-first century

In the format provided by the authors and unedited

Supplementary Discussion 1: Role of remote ocean forcing

Here we estimate the contribution of remote ocean forcing to our simulated warming trends by analysing the Fixed BCs ensembles with present-day climatological boundary conditions. Any significant differences from the main ensembles, which have transient boundary conditions, can be attributed to remote ocean forcing. The main process by which the boundary conditions influence the continental shelf is by changes in the temperature and salinity of inflowing water masses (Methods). For example, surface freshening on the eastern boundary is carried through the domain by the coastal current, stratifies the water column, and causes the thermocline to rise.

Warming of the continental shelf is somewhat faster when transient boundary conditions are applied. The Historical ensemble diverges from Historical Fixed BCs in 1949, and thereafter is slightly warmer (Extended Data Figure 4). However, the differences are initially small enough that the warming trends calculated over the entire historical period are statistically indistinguishable between the two ensembles (Figure 2, Extended Data Table 1). There is also no significant difference from previous historical simulations which additionally constrained tropical Pacific variability[1], reporting an ensemble mean warming trend of 0.33°C/century.

The effect of remote ocean forcing grows over the 21st century, such that warming trends in the RCP 8.5 ensemble are distinct from RCP 8.5 Fixed BCs. Here, the inclusion of transient boundary conditions increases the mean warming trend by 23% and the mean melting trend by 18%. Remote ocean forcing also increases the intra-ensemble variability in warming trends, for both historical and future simulations (Extended Data Table 1).

The effects of remote ocean forcing would be expected to change if CESM1 had a more accurate representation of glacial meltwater. CMIP models, including CESM1, do not typically simulate the Antarctic Ice Sheet (AIS), ice-shelves, or icebergs. Instead, excess snowfall over the continent is rerouted directly to the ocean[2], implying that the AIS has net zero mass balance at all times. In reality, future increases in snowfall should accumulate on the AIS (positive surface mass balance), while simultaneously mass is lost from enhanced ice-shelf melting and iceberg calving (negative basal mass balance). The true net mass balance will depend on which process dominates in which regions of the ice sheet[3]. It is therefore unclear whether CESM1's approach overestimates or underestimates future freshening at the boundaries of our domain. If the freshening were stronger, this would be expected to further stratify the continental shelf and enhance the simulated warming, as well as affect the wider ocean circulation[4] and the global climate[5]. Conversely, if the freshening on the boundaries were weaker, the simulated warming of the Amundsen Sea would be reduced, with a lower bound provided by the Fixed BCs simulations.

Supplementary Discussion 2: Role of convection

The Amundsen Sea warming trend we simulate in the historical period is partially caused by the disappearance of cold convective events on the continental shelf. Here, convection refers to a top-to-bottom mixing of the water column which releases heat to the atmosphere and thus cools the warm CDW layer. It occurs periodically in our simulations through parameterised vertical mixing, and is generally triggered by positive surface salinity anomalies due to sea-ice growth, which destabilise the water column. After a few years, the convection ceases and the warm CDW layer re-emerges.

As discussed by Naughten et al.[1], this model may overestimate the role of convection in the present-day period. While Winter Water has been observed as deep as 700 m in some areas of the Amundsen Sea[6,7,8], true full-depth convection has not been observed. However, such events could have been missed by observations, which only began in 1994 and have since been sparse both temporally and spatially. Nonetheless, given the questions over the realism of convection in the model, it is necessary to quantify the role that it plays in our main results.

Convection occurs intermittently and is an expression of interannual variability. By analysing its occurrence across an ensemble we can estimate the probability of convection in the given forcing scenario. Here we follow Naughten et al.[1] and define convection to occur when the depth of the 0.5°C isotherm, averaged over the Amundsen Sea continental shelf and smoothed using a 24-month running mean, is deeper than a threshold value of 440 m. We then find that convection occurs 10% of the time in the Historical ensemble. This falls to 5% in Historical Fixed BCs, as climatological boundary conditions remove a source of interannual variability in water mass properties. Despite this reduction in the occurrence of convection, the mid-depth warming trends in the two ensembles are not significantly different (as noted in the main text). Convection is also characterised by elevated standard deviation in temperature at depth, as seen for the Historical ensemble in Figure 4b and Extended Data Figure 6. Conversely, when no members are convecting, the standard deviation is near-zero below the thermocline, as seen for the other ensembles.

Convection starts to become less common over the course of the Historical period, as evidenced by the deep warming trend below the thermocline (Figure 4b, Extended Data Figure 6a). This reduction is likely caused by surface freshening stabilising the water column, as in Naughten et al.[1] In the early decades of the future projections, convection disappears completely. For all four future scenarios, convection occurs less than 1% of the time. The final convective events in any ensemble member occur around 2020 in Paris 2°C and 2030 in RCP 8.5 (Extended Data Figure 6). These timings are not inherent features of the forcing scenarios, given the probabilistic nature of convection and the finite ensemble size. Considering reality as a single ensemble member, it is possible that convection is a real phenomenon in the Amundsen Sea, but that it has not occurred during the short observational period, perhaps as a result of historical trends.

Finally, as in Naughten et al.[1], we recalculate the warming trends with convective periods removed. The Historical warming trend decreases by 24%, indicating that the loss of convection plays a secondary but not negligible role in the simulated historical warming. However, the future warming trends are almost unchanged: excluding convection causes the trends to decrease by less than 3% for all four scenarios. This result shows that ocean warming continues through a different mechanism – the increased onshore transport of CDW – long after convection has ceased. We therefore conclude that regardless of whether our simulated convection is realistic, it does not affect our main results.

Supplementary Discussion 3: Analysis of atmospheric drivers

The atmospheric drivers of simulated ocean warming can be surmised by calculating inter-ensemble correlations between trends in atmospheric variables and trends in continental shelf temperature (Extended Data Figure 7). Since the atmospheric fields are not coupled to the simulation, any relationship indicates that the atmosphere is the driver, rather than the response. Previous work proposed that eastward wind anomalies over the continental shelf break drive changes in the undercurrent, both on decadal[9,10,11,12] and centennial[1,13] timescales. However, our simulations display a different relationship: stronger shelf warming trends are associated with cyclonic wind anomalies over the shelf break and deep ocean (Extended Data Figure 7a). This pattern corresponds to westward wind anomalies over the continental shelf, in agreement with Silvano et al.[14], although in this region the correlation is not significant at the 95% level.

Buoyancy fluxes have also been proposed to affect the undercurrent[15,16], as freshening of the continental shelf strengthens cross-slope density gradients and thus speeds up the along-slope flow. Indeed, we find that shelf warming is associated with onshore coastal wind anomalies which reduce sea ice formation and thus cause freshening. We also find a clear relationship with atmospheric thermodynamics, whereby surface warming (Extended Data Figure 7b) and increased precipitation (Extended Data Figure 7c) correspond to ocean warming on the shelf. Averaging over the shelf, there is also a significant correlation with trends in surface freshwater flux. Note that since this variable is affected by sea ice and hence the ocean state, it could be a response to ocean warming as well as a driver.

However, none of these associations can explain the variability in trends within each ensemble. Recalculating the intra-ensemble correlations from Extended Data Figure 7 yields no consistent pattern which is repeated across scenarios. Either internal climate variability is responding to different atmospheric drivers than the scenario response, or the inter-ensemble correlations we find are not causal. We conclude that while ocean warming in our simulations is robust and is primarily driven by a strengthened undercurrent, further experiments would be required to isolate the roles of specific atmospheric drivers.

Supplementary Discussion 4: Model structural uncertainty

Future projection of ice-shelf melting, particularly for the Amundsen Sea, is a relatively new focus for the ice-ocean modelling community. As such, there are no coordinated intercomparison projects analogous to ScenarioMIP[17], which uses CMIP models to examine the wider climate response to plausible future forcing scenarios. The fact that our approach is novel also means that it is subject to model structural uncertainty, as it uses a single ice-ocean model. Currently, the only point of comparison is the RCP 8.5 simulations of Jourdain et al.[18], which used the NEMO model with a time-slice approach and forcing derived from the CMIP5 multi-model mean. They simulated a 40-120% increase in basal melting (depending on parameter choices); using the same region and time periods, we calculate a 100-330% increase (depending on the ensemble member). While these two ranges overlap, the difference in their means highlights the need to better constrain model structural uncertainty related to the underlying ice-ocean configuration. We encourage the community to simulate more future projections of the Amundsen Sea, for multiple forcing scenarios, using a variety of models.

Furthermore, our study uses a single climate model, CESM1, for atmospheric forcing. While a single-model, large-ensemble approach is necessary for clear scenario attribution, it does not provide any estimate of model structural uncertainty in the wider climate system. CESM1 has a climate sensitivity of 4.1°C[19], which is slightly above the CMIP6 multi-model mean of 3.9°C[20]. It is within the 5-95% range 2.3–4.7°C, estimated by Sherwood et al.[21] using observations, paleoclimate records, and process studies. While the climate sensitivity in CESM1 appears to be reasonable, there is still considerable uncertainty in the forcing, which future work could quantify by sampling from across the CMIP ensemble.

Finally, our simulations have static ice-shelf cavities and thus do not consider feedbacks related to ice-shelf geometry. These feedbacks could have a significant impact on basal melt rates[22,23]: for example, thinning ice-shelves expose the cavities to shallower water masses which are generally cooler. While we use buttressing flux response numbers (BFRN) to estimate the sea-level rise implications of our results, this approach does not consider the role of transient ice dynamics which can change buttressing over time[24]. The realism of ocean simulations is also questionable near grounding lines, where BFRN is often highest.

In order to address these uncertainties, it is vital to develop coupled ice-sheet ocean models which allow the ocean cavity geometry to evolve. This approach has been successful in other regions[25] using MITgcm coupled to the ice flow model Úa. The same framework has been achieved technically for the Amundsen Sea, but tuning of the coupled system to prevent unrealistic grounding line drift requires ongoing work. Several additional challenges in the field must be addressed before coupled ice sheet-ocean models can be regularly used for studies such as these. Ice sheet models are generally initialised from observations, so the initial state of a historical ensemble would be almost completely unconstrained. Sea level rise estimates also vary widely between different ice sheet models[26] and are very sensitive to parameterisation choices[27]. While our study focuses on ocean forcing in isolation, future work should assess the impact of this forcing on the ice sheet, considering the additional dimensions of uncertainty.

[1] Naughten, K.A., et al. Simulated Twentieth-Century Ocean Warming in the Amundsen Sea, West Antarctica. *Geophysical Research Letters* 49, e2021GL094566 (2022). doi:10.1029/2021gl094566

[2] Lenaerts, J., et al. Present-day and future Antarctic ice sheet climate and surface mass balance in the Community Earth System Model. *Climate Dynamics* 47, 1367-1381 (2016). doi:10.1007/s00382-015-2907-4

[3] Siahaan, A., et al. The Antarctic contribution to 21st-century sea-level rise predicted by the UK Earth System Model with an interactive ice sheet. *The Cryosphere* 16, 4053-4086 (2022). doi:10.5194/tc-16-4053-2022

[4] Li, Q., et al. Abyssal ocean overturning slowdown and warming driven by Antarctic meltwater. *Nature* 615, 841-847 (2023). doi:10.1038/s41586-023-05762-w

[5] Golledge, N., et al. Global environmental consequences of twenty-first-century ice-sheet melt. *Nature* 566, 65-72 (2019). doi:10.1038/s41586-019-0889-9

[6] Webber, B., et al. Mechanisms driving variability in the ocean forcing of Pine Island Glacier. *Nature Communications* 8, 14507 (2017). doi:10.1038/ncomms14507

[7] Assmann, K., et al. Warm Circumpolar Deep Water at the Western Getz Ice Shelf Front, Antarctica. *Geophysical Research Letters* 46, 80-878 (2019). doi:10.1029/2018GL081354

[8] Steiger, N., et al. Intermittent Reduction in Ocean Heat Transport Into the Getz Ice Shelf Cavity During Strong Wind Events. *Geophysical Research Letters* 48, e2021GL093599 (2021). doi:10.1029/2021GL093599

[9] Dutrieux, P., et al. Strong sensitivity of Pine Island ice-shelf melting to climatic variability. *Science* 343, 174-178 (2014). doi:10.1126/science.1244341

[10] Jenkins, A., et al. Decadal Ocean Forcing and Antarctic Ice Sheet Response: Lessons from the Amundsen Sea. *Oceanography* 29, 106-117 (2016). doi:10.5670/oceanog.2016.103

[11] Kimura, S., et al. Oceanographic Controls on the Variability of Ice-Shelf Basal Melting and Circulation of Glacial Meltwater in the Amundsen Sea Embayment, Antarctica. *Journal of Geophysical Research: Oceans* 122, 10131-10155 (2017). doi:10.1002/2017JC012926

[12] Thoma, M., et al. Modelling Circumpolar Deep Water intrusions on the Amundsen Sea continental shelf, Antarctica. *Geophysical Research Letters* 35, L18602 (2008). doi:10.1029/2008GL034939

[13] Holland, P.R., et al. West Antarctic ice loss influenced by internal climate variability and anthropogenic forcing. *Nature Geoscience* 12, 718-724 (2019). doi:10.1038/s41561-019-0420-9

[14] Silvano, A., et al. Baroclinic Ocean Response to Climate Forcing Regulates Decadal Variability of Ice-Shelf Melting in the Amundsen Sea. *Geophysical Research Letters* 49, e2022GL100646 (2022). doi:10.1029/2022GL100646

[15] Caillet, J., et al. Drivers and Reversibility of Abrupt Ocean State Transitions in the Amundsen Sea, Antarctica. *Journal of Geophysical Research: Oceans* 128, e2022JC018929 (2023). doi:10.1029/2022jc018929

[16] Janzing, J. “Oceanic and Atmospheric Controls on Decadal Ice Shelf Basal Melt Variability around the Amundsen Sea”, thesis, Utrecht University (2022). https://studenttheses.uu.nl/handle/20.500.12932/43356

[17] O’Neill, B., et al. The Scenario Model Intercomparison Project (ScenarioMIP) for CMIP6. *Geoscientific Model Development* 9, 3461-3482 (2016). doi:10.5194/gmd-9-3461-2016

[18] Jourdain, N.C., et al. Ice shelf basal melt rates in the Amundsen Sea at the end of the 21st century. *Geophysical Research Letters* 49, e2022GL100629 (2022). doi:10.1029/2022gl100629

[19] Hurrell, J., et al. The community earth system model: A framework for collaborative research. *Bulletin of the American Meteorological Society* 94, 1339-1360 (2013). doi:10.1175/BAMS-D-12-00121.1

[20] Zelinka, M., et al. Causes of Higher Climate Sensitivity in CMIP6 Models. *Geophysical Research Letters* 47, e2019GL085782 (2020). doi:10.1029/2019GL085782

[21] Sherwood, S., et al. An Assessment of Earth's Climate Sensitivity Using Multiple Lines of Evidence. *Reviews of Geophysics* 58, e2019RG000678 (2020). doi:10.1029/2019RG000678

[22] De Rydt, J., et al. Geometric and oceanographic controls on melting beneath Pine Island Glacier. *Journal of Geophysical Research: Oceans* 119, 2420-2438 (2014). doi:10.1002/2013JC009513

[23] Donat-Magnin, M., et al. Ice-Shelf Melt Response to Changing Winds and Glacier Dynamics in the Amundsen Sea Sector, Antarctica. *Journal of Geophysical Research: Oceans* 122, 10206-10224 (2017). doi:10.1002/2017JC013059

[24] Feldmann, J., et al. Shear-margin melting causes stronger transient ice discharge than ice-stream melting in idealized simulations. *The Cryosphere* 16, 1927-1940 (2022). doi:10.5194/tc-16-1927-2022

[25] Naughten, K.A., et al. Two-timescale response of a large Antarctic ice shelf to climate change. *Nature Communications* 12, 1991 (2021). doi:10.1038/s41467-021-22259-0

[26] Seroussi, H., et al. ISMIP6 Antarctica: A multi-model ensemble of the Antarctic ice sheet evolution over the 21st century. *The Cryosphere* 14, 3033-3070 (2020). doi:10.5194/tc-14-3033-2020

[27] Arthern, R.J. & Williams, C.R. The sensitivity of West Antarctica to the submarine melting feedback. *Geophysical Research Letters* 44, 2352-2359 (2017). doi:10.1002/2017GL072514
